# Modeling homologous chromosome recognition via nonspecific interactions

**DOI:** 10.1073/pnas.2317373121

**Published:** 2024-05-09

**Authors:** Wallace F. Marshall, Jennifer C. Fung

**Affiliations:** ^a^Department of Biochemistry and Biophysics, University of California, San Francisco, CA 94158; ^b^Department of Obstetrics, Gynecology and Reproductive Sciences, University of California, San Francisco, CA 94158; ^c^Center for Reproductive Sciences, University of California, San Francisco, CA 94158

**Keywords:** somatic homolog pairing, polymer dynamics, computational modeling, chromosome dynamics

## Abstract

In many organisms, most notably *Drosophila*, homologous chromosomes associate in somatic cells, a phenomenon known as somatic pairing, which takes place without double strand breaks or strand invasion, thus requiring some other mechanism for homologs to recognize each other. Several studies have suggested a “specific button” model, in which a series of distinct regions in the genome, known as buttons, can associate with each other, mediated by different proteins that bind to these different regions. Here, we use computational modeling to evaluate an alternative “button barcode” model, in which there is only one type of recognition site or adhesion button, present in many copies in the genome, each of which can associate with any of the others with equal affinity. In this model, buttons are nonuniformly distributed, such that alignment of a chromosome with its correct homolog, compared with a nonhomolog, is energetically favored; since to achieve nonhomologous alignment, chromosomes would be required to mechanically deform in order to bring their buttons into mutual register. By simulating randomly generated nonuniform button distributions, many highly effective button barcodes can be easily found, some of which achieve virtually perfect pairing fidelity. This model is consistent with existing literature on the effect of translocations of different sizes on homolog pairing. We conclude that a button barcode model can attain highly specific homolog recognition, comparable to that seen in actual cells undergoing somatic homolog pairing, without the need for specific interactions. This model may have implications for how meiotic pairing is achieved.

## Significance

The physical pairing of chromosomes with their homologs is the basis of Mendelian genetics, but how chromosomes find each other is poorly understood, especially in cases where homolog pairing does not involve breaks in the DNA. We used a polymer model of chromosomes to show that adhesive patches, if distributed in a bar-code pattern, can produce selective homolog pairing even if every patch has the ability to bind any other patch with the same force.

In meiosis, homologous chromosomes are thought to recognize each other at the level of DNA sequence. Specialized enzymes create double strand breaks, from which single strands extend to test homology with other chromosomes. This process of sequence-based homology assessment leads to a highly precise alignment of each chromosome with its correct homolog, allowing for recombination between homologous loci to establish crossovers for proper segregation during meiosis I division. Genetic studies have found that mutations affecting recombination impair homologous association during meiosis in yeast and mice (1, 2), supporting the idea that a DNA-base homology level search is at work.

But while recombination-dependent mechanisms promoting close homolog juxtaposition are clearly important in meiosis, pairing mechanisms that do not require the formation of recombination intermediates contribute to meiotic pairing in multiple organisms, and in some cases, predominate. For example, male meiosis in *Drosophila* involves neither double strand breaks nor recombination, yet homologous chromosomes still associate (3, 4). In *Drosophila* female meiosis, recombination normally takes place during pairing, but if recombination is prevented using mutations, chromosomes still pair and synapse (5). In *Caenorhabditis elegans* (reviewed in ref. 6), homolog pairing does not require recombination (7), and instead, pairing is dictated by chromosome segments known as pairing centers (PCs). When PCs are deleted, pairing is eliminated, and when they are translocated to another chromosome, pairing of that chromosome becomes dictated by the new PC (5, 8, 9).

Even in organisms such as mice and budding yeast, that rely on recombination for full pairing and synapsis, there is evidence that homologous chromosomes are, in some cases, already associated with each other prior to the onset of Double Strand Break (DSB)-mediated pairing (10–16). It has been specifically shown that DSB formation by the *SPO11* enzyme is not required for this pairing to occur (13, 16, 17).

How such recombination-independent pairing mechanisms achieve recognition is not understood. We investigated this question by modeling the process of somatic homolog pairing, where recombination-based mechanisms are not in play. The association of homologous chromosomes in nonmeiotic (somatic) cells is by far the most apparent in dipterans such as *Drosophila*, in which homologous chromosomes are paired in virtually all tissues after the first 13 cell cycles of early embryos (18–21). Somatic pairing has been reported in many different organisms and cell types including in humans (18, 19, 22–24). In some cases, only small chromosomal segments associate with their homologs, and this can vary between cell types or disease states. In some cases, apparent colocalization of loci in nondipterans may result from similar subnuclear positioning rather than pairing per se (25). For this reason, we have chosen to computationally model homolog pairing using parameters roughly appropriate for *Drosophila.* By focusing our analysis on somatic homolog pairing, rather than meiotic pairing, we can avoid the need to represent the complex meiotic processes of DSB-mediated search, recombination, and synapsis.

The physiological purpose of somatic homolog pairing is unknown. In cases where recombination independent association takes place prior to meiosis, it may be involved in setting the stage for a more precise alignment once double strand breaks have formed (26). Somatic homolog association might also facilitate DNA repair by homologous recombination in G1 when sisters are not yet available for this purpose, by placing homologs near each other. In some instances, physical association of chromosomes is involved in trans-regulation of gene expression by regulatory elements located on the other chromosome (27, 28), and pairing may correlate with chromosome functional state (29).

The mechanism of somatic homolog pairing is not known. Genetic analyses of transvection and pairing in flies carrying translocations and other chromosome rearrangements have shown that large chromosome regions, rather than specific DNA elements such as enhancers or insulators, are involved in assessing homology (30–32). This has led to the idea of a “Specific Button” model, in which chromosome regions sparsely distributed along chromosome arms mediate specific associations (32). This Specific Button model is consistent with both FISH (21) and live-cell imaging (33) studies of somatic pairing in *Drosophila*, which showed that chromosomes do not “zip up” continuously along their length, but instead initiate pairing independently at multiple distinct regions.

**Fig. 1.**
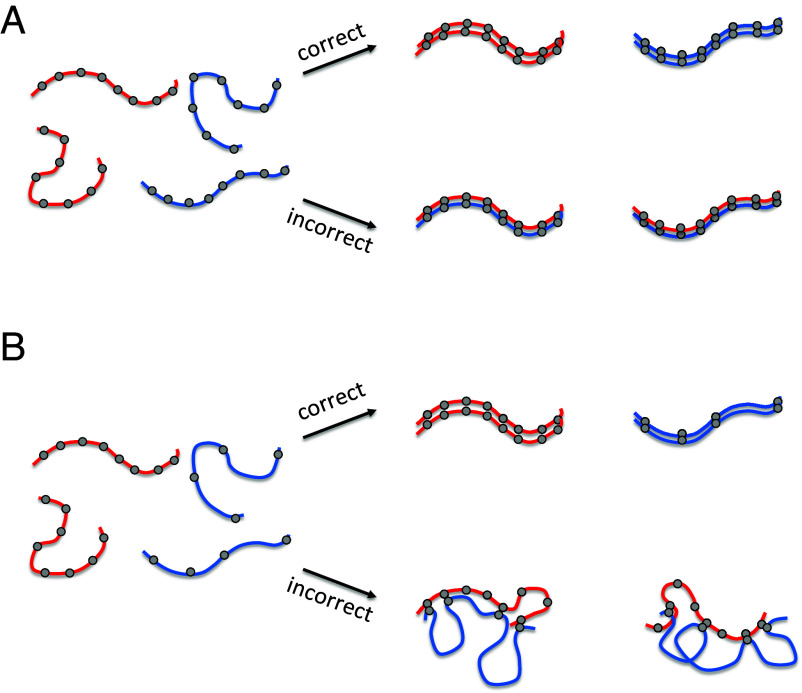
A button barcode model for homolog recognition by nonspecific interactions. (*A*) Uniformly spaced nonspecific pairing buttons. In this case, gray circles denote pairing buttons, each of which has the same molecular affinity for every other such button in the genome. Two different pairs of homologs are denoted by red and blue color. Correct pairing (red pairs with red, blue pairs with blue) and incorrect pairing (red pairs with blue) would each be equally likely. (*B*) Button bar-code model, in which nonspecific pairing buttons have different spacing patterns on the two chromosomes. In this case, pairing with the incorrect homolog incurs an energetic cost for deforming one or both chromosome polymers so as to allow the buttons to physically interact. Pairing with the correct homolog does not require such deformation and is thus energetically favored, hence more probable.

In a recent tour-de-force study of the kinetics of somatic pairing in *Drosophila*, Child et al. (33) implemented a computational version of the button model for pairing, in which a set of discrete pairing sites distributed at regular intervals along the chromosome could engage in independent pairing interactions that, collectively, would align the two homologs along their length. Computational modeling indicated that this model can account for pairing kinetics consistent with live cell rate measurements, but it only works if the buttons are distinct, in the sense that a button at a given position on a chromosome can only pair with a corresponding button on the homologous chromosome. A model in which the buttons lacked specificity was not able to achieve homologous recognition (33). It has been shown that regularly spaced nonspecific association sites are able to at least bring chromosomes into alignment, but again without any specificity (34). The lack of specificity is simply because there is no energetic difference between associating with the correct vs. incorrect pairing partner (Fig. 1*A*).

Inspired by the work of Viets (32) and Child (33), we investigated a variant of the button model, in which the pairing “buttons” are individually nonspecific, such that every button is equally capable of pairing with any other button, but in which the buttons are nonuniformly arranged in a different pattern on different chromosomes, allowing them to act as a code specifying chromosome identity (Fig. 1*B*). We will refer to this model as the “Button Barcode” model. Specificity of homolog pairing requires the chromosome pairing process to be able to distinguish the spacing of these nonspecific buttons over a potentially long spatial scale. We propose that the physics of the chromatin polymer will tend to favor association of buttons that are equally spaced on both chromosomes. Association of pairs of buttons with different spacings on two nonhomologous chromosomes will require one or both chromosomes to either stretch or compact, incurring a mechanical energetic cost. In such a model, the information about chromosome identity is encoded in the spacing between buttons, in much the way that an industrial barcode encodes information in the spacing between bars printed on a package (35). The key feature of this model is that the spacing between the buttons, not the position of buttons per se, is the origin of selective association.

Here we use a coarse-grained computational model of chromatin to investigate the plausibility of this button barcode model. Our model is not intended to represent any particular species or model system, but just to reflect generic properties of chromosome polymers. We show that unequal spacing of nonspecific interaction sites on different chromosomes is in fact sufficient to produce a reliable association of chromosomes with their homologous partners. We show that this specificity depends on the mechanics of the chromosomes; on the three-dimensional organization of chromosomes within the nucleus, specifically the Rabl configuration in which centromeres cluster at one end of the nucleus and telomeres at the other; and on the reversibility of pairing interactions. We show that randomly spaced buttons are able to achieve a level of specificity that matches what is seen in actual cases of somatic pairing. Finally, we implement a chromosomal version of a standard industrial barcode known as “code 2 of 5” (36) and show that it outperforms many random button patterns. Our results show that, at least in principle, sequence-level specificity is not required for accurate homologous pairing, and suggest some features that would be required for this type of mechanism to work. We discuss this model in light of evidence for and against specific buttons, and conclude with a model in which barcode segments built from short tracts of nonspecific interaction buttons can act like specific buttons at a larger scale, thus potentially explaining the existing data concerning the effect of translocations on pairing while avoiding the need to posit specific interactions at a molecular level.

## Results

### Modeling Somatic Chromosome Interactions.

The essence of the Button Barcode model is described in Fig. 1*B*. In this model, any button can pair with any button, and selectivity for the correct homolog is a consequence of the different arrangement of the buttons along the different chromosomes combined with the physics of the chromatin polymer. Although chromatin is sometimes modeled as a freely jointed random chain, actual chromosomes are in many cases better described as worm-like chains or other forms of elastic polymers, in which energy is required to bend them away from an equilibrium (37–40). The end-points of any segment of such a worm-like chain will have a characteristic distribution of lengths that is energetically favored. Trying to move the ends of that segment closer together or farther apart will incur an energetic cost. Because of this energic cost to deforming (either looping or stretching) the polymer, a side-by-side alignment of actual homologs should be energetically favored over alignment of nonhomologs, because only an association of actual homologs allows the segments between adjacent pairs of buttons to remain at their energetically favored lengths. If nonhomologs attempt to associate, it would require a bending or stretching of one or both homologs in order to accommodate the mismatch in spacing between adjacent buttons.

**Fig. 2.**
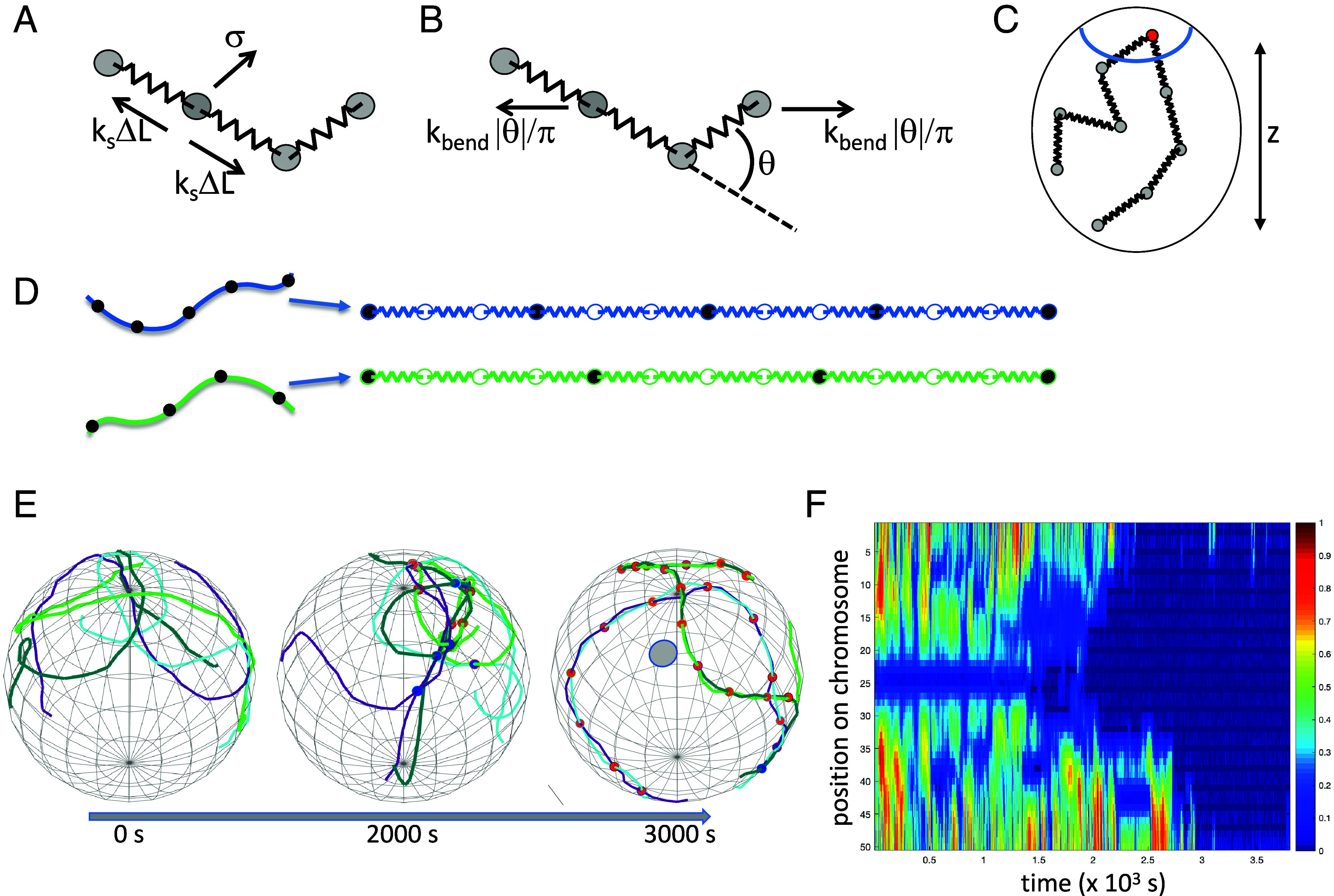
Computational model for somatic homolog pairing. (*A*) bead-spring model in which the chromosome is represented by beads linked by Hookean springs with spring constant k_s_. Each bead is subject to a random Langevin force σ, as well as forces generated by the springs. The entire chain moves in a medium with a specified frictional coefficient. (*B*) Chromosome flexibility modeled using a worm-like chain model in which adjacent nodes are pulled apart by a force proportional to the deviation of the chain shape from a straight line. The higher the bending constant, the less flexible the chain. (*C*) Chromosomes are confined within a spherical nucleus. Centromeres, indicated by the red node, are attached to the nuclear envelope within a confined surface patch denoted by the blue circle, creating a Rabl orientation and defining a vertical (z) axis for the nucleus. (*D*) Representation of button barcode in the bead-spring model. For each bead-spring chain representing a chromosome, a subset of nodes are defined to be adhesive buttons and are allowed to reversibly pair with any other adhesive buttons. The example in this panel shows part of two chains, one in which the buttons are located every three nodes, and one in which the buttons are located every four nodes. (*E*) Image sequence from a representative simulation with buttons present at a regular but different spacing on the two chromosomes, with buttons located every three nodes on one chromosome and every four nodes on the other, spanning the entire chromosome. Two pairs of homologs were simulated, with one pair of homologs plotted in dark and light green, and the other in cyan and purple. Incorrectly paired loci are marked with blue spheres, and correctly paired loci are marked with red spheres. (*F*) Pairing kymograph plotting distance between homologous loci for each position along one chromosome. X and Y axes denote timesteps of simulation and position along chromosome, respectively. Color shows the distance of each locus to its homolog, normalized to the maximum distance between all homologs observed in the simulation, with red indicating maximum distance, and dark blue representing zero distance (corresponding to the paired state). The color bar gives the colors as a function of fraction of maximum distance.

In order to test the plausibility of this model, we implement a Brownian dynamics simulation based on prior modeling of meiotic chromosome movement and pairing (41, 42). As illustrated in Fig. 2*A*, we represent the chromosome using a bead-spring model, with each node (bead) subject to a Langevin random force that represents thermal energy, as well as forces applied by the springs linking that node to its two neighbors. We also impose a series of torsion spring forces that tend to push the chromosome toward an elongated linear form, creating a worm-like chain model (Fig. 2*B*). The bead-spring chains are confined to a spherical nucleus, within which centromeres are clustered at one end of the nucleus (Fig. 2*C*) to mimic the Rabl configuration in *Drosophila*, where centromeres and telomeres are at opposite ends of the nucleus and centromeric heterochromatic is tightly associated with the nuclear envelope (43). Finally, a subset of nodes in the chain are defined to be adhesive buttons, any of which can associate with any other, and whose distribution along the chain reflects the button barcode for that chromosome (Fig. 2*D*)

Fig. 2*E* shows three time points from a representative simulation, in which the buttons are spaced at regular intervals on both chromosomes, but with different spacings (three nodes apart on one chromosome, four nodes apart on the other). Early in the simulation, chromosomes have not yet paired. Later, buttons have begun to associate with each other, some with the correct (homologous) button, but others with incorrect buttons. This is not surprising given that there is no selectivity in terms of which buttons are allowed to pair with other buttons. As the simulation proceeds, fewer and fewer incorrect associations are seen, and more and more correct associations are seen. A pairing kymograph plot (41), which depicts the distance of each locus to its homolog over time, shows that a set of completely nonspecific buttons, arranged with unequal spacing on the two chromosome pairs, ends up producing close association of all loci with their homologous loci (Fig. 2*F*).

In this particular simulation, pairing was achieved within approximately an hour of simulated time. This timescale is consistent with measurements in *Drosophila* embryos, which have shown that the histone locus in *Drosophila* achieves as much as 80% pairing in the first 20 min of cycle 14 (21), but other loci pair more slowly, with fraction of pairing increasing gradually over a 6 h period starting at the beginning of cycle 14 (21, 33).

We note that while our model is roughly based on observations of somatic homolog pairing in *Drosophila* embryos, the model itself is highly simplified and is not meant to represent any particular species or cell type. Instead, the goal is just to test the plausibility of such a model and explore what features a chromosome would need to have in order to achieve a sufficiently high degree of correct homologous pairing.

### Influence of Polymer Mechanics on Pairing Specificity.

**Fig. 3.**
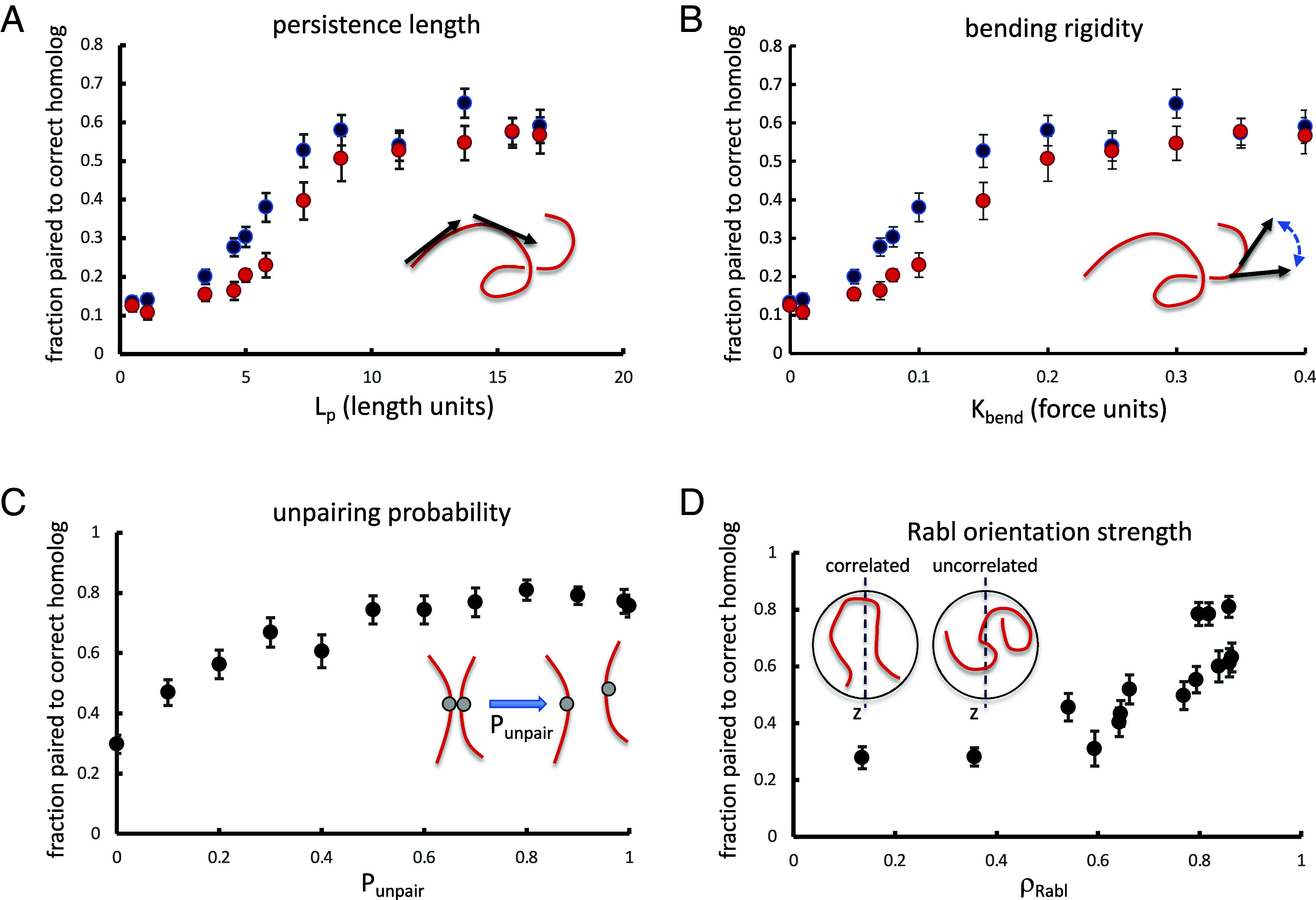
Influence of chromosome polymer physics and nuclear organization. (*A*) Effect of chromosome flexibility as quantified by persistence length. The plot shows the results of simulations of pairing of two five-button tracts with different spacing (3 vs. 4 nodes between buttons), plotting the fidelity, defined as the fraction of nodes paired to their correct homolog, vs. persistence length. The *X* axis is persistence length in simulation length units. Two sets of simulations were run, one in which the two tracts were centered on the same node in both chromosome pairs (blue), and the other in which the tracts were offset by 11 nodes between the two homolog pairs (red). *Inset* illustrates the definition of persistence length as the distance along the polymer over which the orientation of the chain, marked with black arrows, becomes uncorrelated. (*B*) Data from panel *A* replotted as a function of the bending rigidity, kbend, which was the parameter used in our simulations to alter the persistence length in panel *A*. *Inset* illustrates the definition of kbend as a force that resists change in the orientation of the polymer chain between adjacent positions, such that higher values cause the polymer to straighten out more. (*C*) Effect of reversibility of pairing. Simulations were carried out using a uniform spacing between buttons (3 vs. 4 nodes between buttons on the two chromosomes) with buttons spanning the whole arm, but with different values of p_unpair, the probability that two paired buttons will become unpaired at each timestep. *Inset* illustrates the definition of p_unpair as the probability that two loci, currently paired, will become unpaired during one iteration of the simulation. (*D*) Effect of Rabl orientation. The plot shows the results of simulations as in panel *B*, but in which the confinement radius for centromere clustering was decreased to reduce the strength of the Rabl configuration. The *X* axis is the Rabl correlation coefficient, defined as the correlation between genomic position (distance from centromere) and vertical position (along the *z* axis of the nucleus). *Inset* illustrates definition of Rabl orientation strength as correlation of position along chromosome way from the centromere with the *z*-axis position of the locus within the nucleus. Simulations for all panels in this figure were all run for 300,000 iterations per simulation run. All simulation results plotted are the average of 30 separate simulation runs. Error bars represent SEM.

We next investigated the influence of several key model parameters on the ability of the button barcode model to give correct homolog pairing. All of these simulations, summarized in Fig. 3, used buttons spaced at regular intervals in which the spacing between buttons was different on the two different chromosomes (details are provided in the figure legend) We modeled two sets of homologous chromosomes. We assess pairing fidelity in terms of the fraction of loci that are paired to loci on the correct homologous chromosome. Thus perfect fidelity would be reflected as 100% pairing to the correct homolog. For every chromosome, there is one correct homolog that it should pair with, but three incorrect chromosomes that it should not pair with (i.e., either of the two nonhomologs also present, or else anywhere else on its own chromosome, i.e., pairing in cis). In the absence of any mechanism to promote pairing fidelity, one would expect to observe around 25% correct pairing just based on chance alone.

Our model for chromosome dynamics treats the chromosome as a worm-like chain, which can be characterized by the persistence length, a standard way to quantify the flexibility of a polymer in terms of the length scale over which the orientation of the polymer becomes uncorrelated. For a freely jointed polymer, with no restriction on the bending angle at each node in the chain, the persistence length would be half the link length of the chain (44) corresponding to 0.5 length units. In the button barcode model, the higher the persistence length, the greater the energetic penalty for associating buttons with different spacings on their respective chromosomes, and therefore the greater expected fidelity of pairing. As shown in Fig. 3*A*, this is indeed the case—as the persistence length is increased (*Materials and Methods*), the fidelity increases up to a plateau value. On the other hand, as persistence length decreases, the fidelity also decreases, down to a minimum when the persistence length is that of a freely jointed random coil. For such a freely jointed random chain, the fraction of loci paired to the correct homolog is actually less than the theoretical minimum of 25%. We interpret this to mean that for a highly flexible chain, a given locus can pair not only with its correct homolog plus two incorrect homologs, but also to other loci on the same chain, thus giving it more incorrect options, and that for a random coil, self-association might become highly favored due to the more compact shape of the folded coil compared to a more extended worm-like chain. We note that the way we changed the persistence length in these simulations was to change the bending rigidity parameter kbend (see *SI Appendix* and *Materials and Methods* for details of model parameters). Fig. 3*B* plots the same data as panel A, but as a function of kbend.

In these simplified modeling studies, we simulated persistence lengths in the range from 0.5 to 16.7 length units, corresponding to 0.1 to 3.3 µ. We found that correct associations occur frequently when the persistence length exceeds roughly 5 length units, which corresponds to 1 µ (Fig. 3*A*). This is much longer than the persistence length of 50 to 80 nm for DNA reconstituted with nucleosomes (45), but is only several fold higher than the persistence length of 220 nm reported for yeast interphase chromosomes (39).

Is this persistence length range plausible for *Drosophila* embryos, the system we are modeling for somatic homolog pairing? Chromatin persistence length has not, to our knowledge, been directly measured during interphase in *Drosophila* embryos. But persistence length has been measured for mitotic chromosomes in *Drosophila* embryos, using 3D imaging in live embryos, which were shown to have a persistence length of 150 µ (46). Thus, the maximum persistence length we used in our simulations, while higher than that of isolated nucleosomal fibers, is 50-fold less than that of a mitotic chromosome, while the standard value we used for most simulations of 1 µ is more than 100-fold less. In *Drosophila* embryos, interphase chromosomes are compacted 20-fold relative to the presumptive 30 nm fiber (47), which is approximately 10-fold less than the compaction of a mitotic chromosome (48). Given that a less compact chromosome will have a proportionally lower elastic modulus and thus a shorter persistence length, the range of persistence lengths used in our simulations is well within the range one would expect given the level of decompaction seen in this organism.

We also compared pairs of tracts that were located at the same distance from their respective centromeres, with pairs of tracts that were offset with one located near its centromere and the other located near its telomere (blue vs. red markers in Fig. 3*A*). Both arrangements showed the same general trend that increasing persistence length increased pairing fidelity, but higher fidelity was seen when the tracks were offset, which we interpret as an outcome of the Rabl configuration, which would tend to disfavor association of buttons located at different distances from their centromeres.

### Influence of Pairing Reversibility on Pairing Specificity.

We next consider the influence of unpairing probability on the achievement of high-fidelity pairing. Previous simulations of a specific button model (33) considered only irreversible pairing, which is appropriate for a model in which the on-rate for pairing is highly selective, to the extent that only correct (i.e., corresponding to identical loci on the correct homologs) buttons are allowed to pair in the first place. In our model, however, since button associations are completely nonspecific, irreversible pairing would be expected to lock in incorrect associations, preventing them from ever being corrected. We therefore carried out simulations using a range of values for the parameter p_unpair, which describes the probability that two paired buttons might become unpaired during one iteration of the simulation. We have previously shown, in models with selective pairing sites, that high values for unpairing probability can still allow homologs to form stable associations (42). Here, we investigate the effect of unpairing on the nonspecific button barcode model. Fig. 3*C* shows the fraction of loci paired to the correct homolog as a function of p_unpair. The worst performance is when p_unpair is zero, that is, when pairing is irreversible. This result is consistent with our intuition that reversible pairing is needed to correct errors in association, and also matches our observations on simulations in which many incorrect associations can be seen at early time points, which are later corrected (see for example Fig. 2*E* second time frame). In these simulations, maximum fidelity of pairing is achieved at a value of p_unpair of 0.8, but the actual maximum fidelity is a function of the specific barcodes chosen. Further increase in unpairing leads to a decrease in pairing, but it is interesting to note that even at a value of 1.0, meaning that every pair of loci will unpair at each iteration, homologous association still takes place. The reason for this effect is that when two buttons unpair, they remain near each other, and are thus biased to rapidly reassociate in the next time point. In any case, the main conclusion is that reversibility of association is not only tolerated, but is actually essential for selectivity in homolog recognition by nonspecific buttons. The importance of pairing reversibility has been previously discussed in the context of meiotic chromosome pairing (49) and has been directly observed during meiosis in living yeast cells (16). It is therefore plausible that pairing for somatic chromosomes would be similarly dynamic.

The fact that higher unpairing rates give better pairing in our model suggests that homologs are most effectively recognized by multiple weak interactions. In *SI Appendix*, Fig. S1, we calculated the energetic cost of incorrect vs. correct pairing, and found that this energy of discrimination, created by the energetic cost of deforming the chromosomes to allow pairing of unequally spaced buttons, is on the order of several k_b_T, confirming that the interaction energy is relatively weak.

### Influence of Large-Scale Nuclear Architecture on Pairing Specificity.

Chromosomes are not, in general, randomly arranged in nuclei (50, 51). One of the best-understood aspects of nuclear organization is the Rabl orientation, in which interphase chromosomes retain a vestige of their orientation from anaphase, such that the centromeres colocalize at one end of the nucleus, and the telomeres at the other, with the chromosome arms stretching in between. This configuration is seen in many different species and cell types (50, 52–55), but is perhaps most dramatically seen in *Drosophila* embryos (43, 56), where centromeres cluster at one end of the nucleus closest to the embryo surface and telomeres are at the other end of the nucleus. In *Drosophila* embryos, each chromosome arm contains 25 Mb of DNA and spans the length of the nucleus, which is 4 µ in cycle 13 but increases to more than 12 µ in cycle 14 when somatic pairing initially becomes most apparent.

One effect of the Rabl orientation is that corresponding loci on homologous chromosomes will tend to be nearer to each other than randomly chosen nonhomologous loci, because they are the same genomic distance from their corresponding centromeres. Referring to the centromere–telomere axis as the vertical axis of the nucleus, these loci can be said to have similar vertical positions. This might give them a stronger tendency to associate with each other than with loci at other genomic locations, which would thus lie at different vertical positions.

In order to test the effect of the Rabl configuration on pairing fidelity, we performed a series of simulations using button tracts spanning the whole chromosome arm, with a spacing of 3 nodes between buttons on one chromosome and 4 on the other, in which we progressively reduced the degree of centromere clustering by increasing the diameter of the region in which the centromeres were confined. For each confinement region, we first ran the simulation without pairing and calculated the correlation coefficient between position on the chromosome and position along the z axis, which we take as a measure of the strength of the Rabl configuration. We then performed pairing simulations and plotted the average pairing fidelity vs. the strength of the Rabl configuration. As shown in Fig. 3*D*, the best pairing was obtained with the strongest Rabl orientation, and when the Rabl configuration was reduced to the point that chromosomal position and vertical position were uncorrelated, the pairing fidelity dropped to near the theoretical minimum value of 25%.

### Homology Recognition Using Randomly Generated Button Codes.

Thus far, we have only considered the case in which different homologs have different, but uniform, spacing between their nonspecific interaction “buttons.” We have shown that such a scheme can indeed lead to a majority of chromosome loci associating with the correct homolog compared to an incorrect homolog on which the buttons have a different spacing. But this scheme was arbitrarily chosen as a proof of concept, and we have no reason to believe it is the best possible way to achieve homolog discrimination. For one thing, by having regular spacing between all buttons on a chromosome, there is a potential for the pairing to get out of register, such that button n associates with button n+1 on the homolog. Such out of register association with the correct homolog would not incur an energetic penalty in that neither homolog would be required to stretch or bend to achieve alignment. A second limitation of regular spacing is that different regular spacings on different chromosomes requires different densities of pairing sites on different chromosomes, which may or may not be biologically desirable. More generally, if we want to use our theoretical work as a source of hypotheses about the possible distribution of pairing sites in actual chromosomes, it is important to have an idea of what pairing site distribution is optimal, under the assumption that selection pressure may have driven a similar distribution in real chromosomes.

**Fig. 4.**
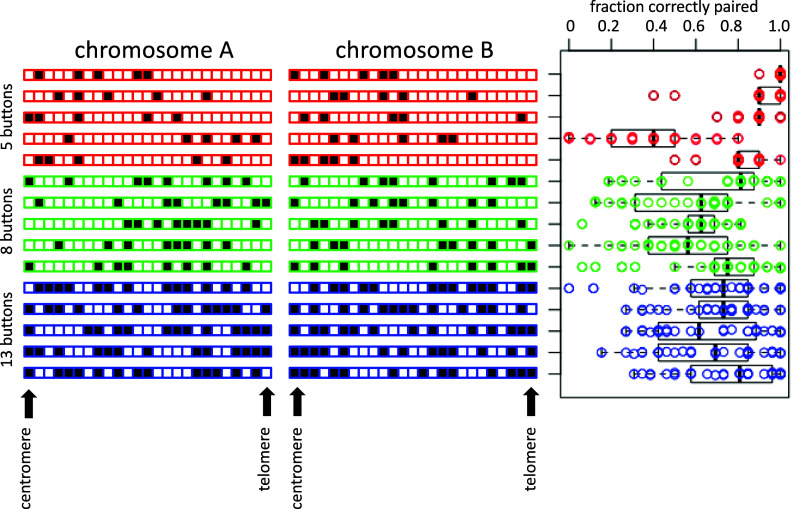
Randomly generated coding can achieve selectivity. Bar diagrams show the location of pairing buttons on the two chromosomes, with each square corresponding to a node on one chromosome arm, and a block box corresponding to a node with a pairing button. Red, green, and blue illustrate randomly generated arrangements of 5, 8, and 13 buttons, respectively. The beeswarm plot shows the pairing outcomes (fraction of loci paired with the correct homolog) for 30 simulations of each random code pair. Box plots were made using the boxplot function in r, such that vertical bars indicate median, boxes indicate second and third quartiles (25 to 75% of datapoints), and whiskers represent 1.5 times the interquartile range. This same boxplot method is used in all subsequent figures. In each case, the theoretical minimum pairing fidelity if all buttons were pairing nonselectively and independently of each other, is 0.25. The simulations in this figure, and all subsequent figures, were each run for 300,000 iterations.

In order to see how the pattern of buttons along a chromosome might influence the fidelity of homolog recognition, we generated a series of random button distributions and simulated pairing in each case. As shown in Fig. 4, when we generate random codes at three different densities of buttons, we observed a range of pairing fidelity. The majority of random codes were able to give pairing fidelity greater than 60%, which is comparable to the level of correct somatic homolog pairing in many actual cases (see below). In one case, the fidelity was extremely high (>99%). The fact that almost perfect homolog pairing could be obtained just by sampling a small number of random codes suggests that a button barcode could be easy to evolve.

**Fig. 5.**
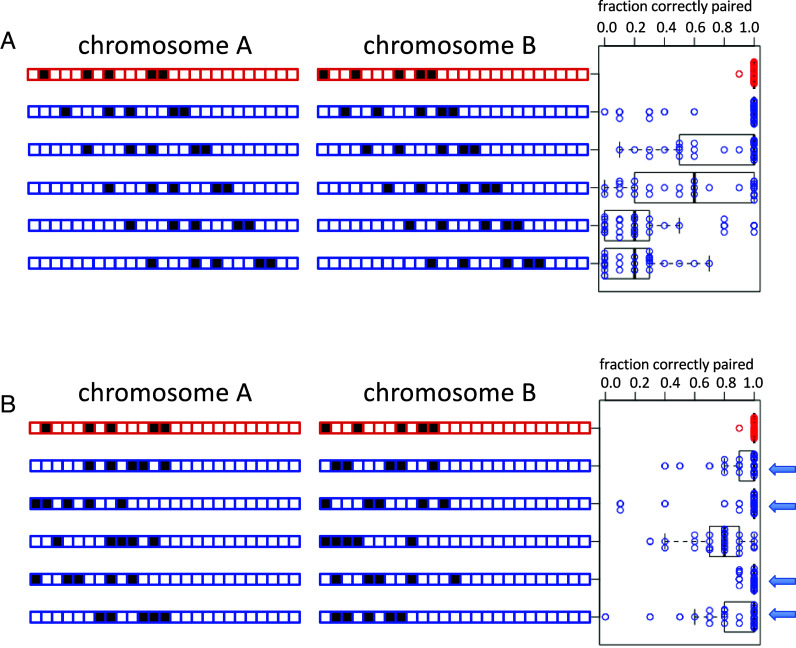
Variations on the five button randomly generated code. (*A*) The best-performing randomly generated code from Fig. 4 was shifted progressively away from its centromere in intervals of two nodes. The red bar illustrates the original location of the buttons. For each shifted version, the bar plot displays the location of the buttons. The beeswarm plot shows the pairing outcomes from 30 simulations for each code pair. (*B*) Testing other randomly generated five button codes constrained to span the same range of nodes as the optimal five button code from Fig. 4 (which is shown in red). Four of the five random code pairs (indicated by arrows) give essentially as good pairing outcomes as the original code pair.

Looking at the five-button random codes, the code pair that gave the best performance was one in which the buttons on both chromosomes were clustered near their corresponding centromeres. We hypothesized that part of the reason these codes worked so well might be that their proximity to the centromere makes them maximally subject to the Rabl constraint, since in our model, this constraint was implemented solely by clustering centromeres together. To test this idea, we repeated the simulation of the same pattern of buttons, shifting the pattern progressively away from the centromere. As shown in Fig. 5*A*, the fidelity of pairing decreased continuously as the tracts were moved away from the centromeres, consistent with the idea that the button barcode segments work best when located near clustered centromeres.

Based on these results, we generated a further set of random five-button barcodes, this time constraining them to all lie within a 13 node stretch of the simulated chromosome at the end near the centromere. As shown in Fig. 5*B*, four of the five additional random five button codes (marked with arrows) were also highly effective for chromosome pairing, comparable to the original high-performing random five button code from Fig. 4.

We conclude that random arrangements of nonselective buttons positioned with nonuniform spacing can in fact lead to extremely efficient homology recognition. However, among the randomly generated nonuniform button patterns, the ones that work the best seem to be those that are restricted to subregions of the chromosome arm.

In an effort to further explore the variety of possible button barcodes, we next turned to actual barcodes encountered in everyday life.

### Homology Recognition Using an Industrial Barcode.

Barcodes are familiar in our everyday lives, printed on virtually all commercial products. Barcodes are patterns of black vertical stripes separated by white vertical spaces (35). Using just these two colors, it is possible to discriminate a large number of different symbols, based on the pattern of widths of the stripes and spaces (57). Most bar codes, such as UPC or Code 39, encode information in the widths of both the white and black bars. This is different from the chromosome pairing code we propose here, in that in the chromosome pairing case, only the spacing between the pairing sites encodes information by affecting the energy required to simultaneously pair buttons on either side of the gap, and all the pairing sites themselves are treated as equivalent (none of the buttons are viewed as longer or stronger than any others). This would be analogous to a barcode in which all the information is encoded by the widths of the bars, but in which all spaces have equal length. In fact, such barcodes do exist, the most common example being code 2 of 5 (35).

A second feature of real barcodes is that in general the width of the stripes is constrained to take on just one of two values, such that there are wide stripes and narrow stripes and no other options. For most bar code symbologies, the wide stripes are three times wider than the narrow stripes, with the widths selected so as to maximize the difference between wide and narrow stripes, while subject to constraints concerning the minimum size of the narrow stripe and the total number of stripes in the coded character (35). In a few rare examples, such as the Codabar barcode, the bars are not multiples of a unit width and multiple different bar widths end up appearing within a code (36), however this variation in bar widths ends up not increasing the information capacity, and such codes can be replaced with variants using just two bar widths (58).

A third feature of real barcodes is that the order in which the bars occur is critical—if the bars are read in a different order, the symbol will be decoded differently. This is generally not an issue in real barcodes that are printed onto a rigid surface, but for a chromosome bar code, there is certainly the potential for bars (which we now interpret as the spacing between successive pairing sites on a chromosome) to be read out of order, depending on how the chromosome polymers are folded. Our data above showed that reliable decoding (i.e., choosing the correct pairing partner) requires that chromosomes are not just random coils, but maintain some linear structure due to the physics of a worm-like chain. The more rigid the chain, the more the arrangement of spacings between pairing sites will approximate a real barcode printed on a solid surface.

Finally, during the decoding of a real barcode, the symbol is scanned from one end all the way to the other. This allows the bars to be read in the correct order, which essentially means that as each bar comes up, it can be compared computationally to an internally stored reference pattern. In the case of the chromosome button barcode, decoding the bars in the correct order is enforced by the Rabl orientation.

**Fig. 6.**
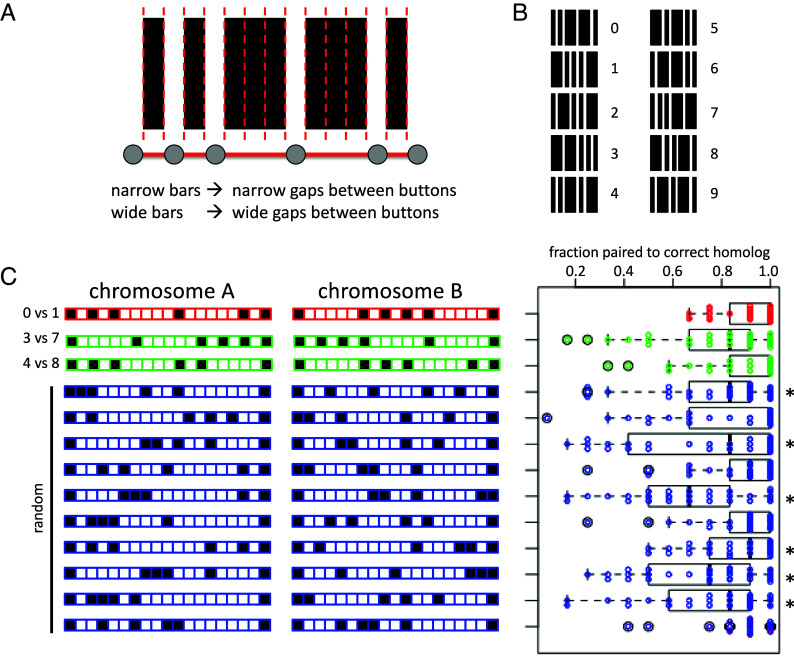
An industrial barcode can achieve pairing selectivity. (*A*) Analogy between an industrial barcode and the pattern of chromosome button spacing. Buttons on the chromosome correspond to the white bars in the industrial bar code, which are all identical. Gaps between buttons correspond to the width of the black bars in the industrial barcode, which take on two values, narrow and wide. (*B*) The “code 2 of 5” barcode symbology (58). Information is only encoded in the width of the black bars, while the spacing between the bars carries no information. Every character consists of five bars, two wide and three narrow. (*C*) Button barcode derived from 2 of 5 by setting each narrow bar equal to a gap of two nodes between buttons, and each wide bar equal to a gap of 6 nodes between buttons. Beeswarm plots show the outcome of a 2 of 5 code using symbols for 0 and 1 (red), 3 and 7 (green) and 4 and 8 (green), compared to random simulations with the same number of pairing sites distributed with the same endpoints (blue). Asterisks denote random codes giving significantly less correct pairing compared to the 2 of 5 barcode simulations of 0 vs. 1, at a significance of 0.05 or better (one-tailed Mann–Whitney test).

There is thus a very concrete analogy between real bar codes and chromosome pairing barcodes, suggesting it would be possible to encode an actual barcode on a chromosome (Fig. 6*A*). To do this, we start with the 2 of 5 code (36) in which a) all information is encoded by the widths of the black bars, b) there are just two possible widths for the black bars, with the wide bars being three times as wide as the narrow bars, c) all white bars are the same width corresponding to the narrow black bars, and d) every character is encoded by five bars, of which two are wide. This code was designed to represent the digits 0 to 9 (Fig. 6*B*). To generate a chromosome pairing code based on 2 of 5 code, we treat the pairing buttons on a chromosome as the white bars in code 2 of 5, and the gaps between pairing buttons as the black bars. To achieve the 3:1 ratio of wide to narrow stripes in code 2 of 5, while spanning most of one chromosome arm, we space buttons 6 nodes apart for a wide bar and 2 nodes apart for a narrow bar. Fig. 6*C* shows the chromosome implementation of two different digits, 0 and 1, in 2 of 5 code. As seen in Fig. 6*C*, chromosomes printed with 2 of 5 code can be discriminated with a reliability of 0.91.

For comparison, we also simulated random codes with the same number of buttons as the 2 of 5 code and spanning the same range of nodes, with buttons fixed at the same endpoints. While several of the random codes performed almost as well as 2 of 5 in terms of their average fidelity, the 2 of 5 barcode simulation showed a clear difference in terms of the left tail of the distribution—in comparison to the random codes which sometimes gave very poor results, 2 of 5 never had less than 60% match to the correct homolog. This suggest that the industrial barcode performs better than comparable random patterns in terms of minimizing worse case results.

### Pairing in the Presence of Multiple Chromosomes.

**Fig. 7.**
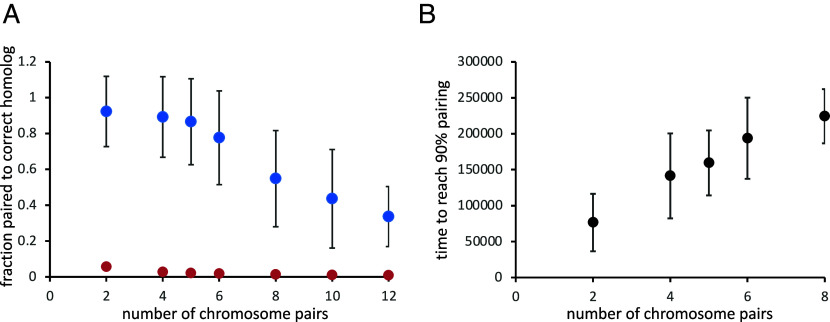
Pairing of multiple chromosomes. (*A*) Results of simulations using different numbers of chromosomes, with each additional pair containing a different random five-button code. (blue) Average fraction of buttons that have paired to a corresponding button on the correct homolog. (orange) Predicted fraction of correct pairing if buttons randomly associate with other buttons. The *X* axis indicates the number of chromosome pairs. Error bars represent SD for 30 simulations. (*B*) Time to reach full pairing vs. number of chromosome pairs. Pairing time was scored based on the number of simulation timesteps until at least 90% of buttons were paired with the correct homolog. Pairing times were not calculated for more than eight chromosome pairs because 90% pairing was never reached in those simulations.

The simulations thus far only represented two pairs of homologs, in order to ask whether nonspecific buttons could, in principle, allow a correct homolog to be distinguished from an incorrect chromosome. However, most actual cells have more than two pairs of chromosomes, leading us to ask how this nonspecific button barcode performs when more chromosomes need to be discriminated. As shown in Fig. 7*A*, when we expand the simulation to include more chromosome pairs, random barcodes still provide high levels of correct pairing with as many as 5 or 6 chromosome pairs, but then pairing efficacy falls off as the number increases beyond 8. The poorer performance with increasing chromosome numbers is also reflected in the time required to achieve 90% pairing, which, as plotted in Fig. 7*B*, increases approximately linearly with chromosome number over the range of 2 to 8 chromosome pairs. Recognizing the limitations of the current simplified model, these data suggest that a nonspecific button barcode might work best when the number of chromosomes are small. In this respect it is interesting to note that *Drosophila* only has four chromosome pairs (X, 2, 3, and 4), which at least in our simulations is a small enough number to allow rapid and specific homolog pairing. These results may suggest that the decreased levels of somatic homolog pairing seen in other organisms might result in part from their larger number of chromosomes or larger genomic content.

Although complete pairing decreases with larger number of chromosomes, the fraction of correctly paired buttons is still vastly greater than what would be expected by purely random associations, as indicated by the orange markers in Fig. 7*A*. These simulation results thus predict that a nonspecific barcode based on random but different arrangements of buttons on different chromosomes would have difficulty achieving complete pairing at all sites when the number of chromosomes is large, but would still be able to cause a strongly nonrandom association between homologs, potentially resulting in an overall alignment that could, in the context of meiosis, accelerate subsequent homology searching by more specific mechanisms such as by the recombination machinery.

### Homolog Recognition by Short Barcode Patches.

**Fig. 8.**
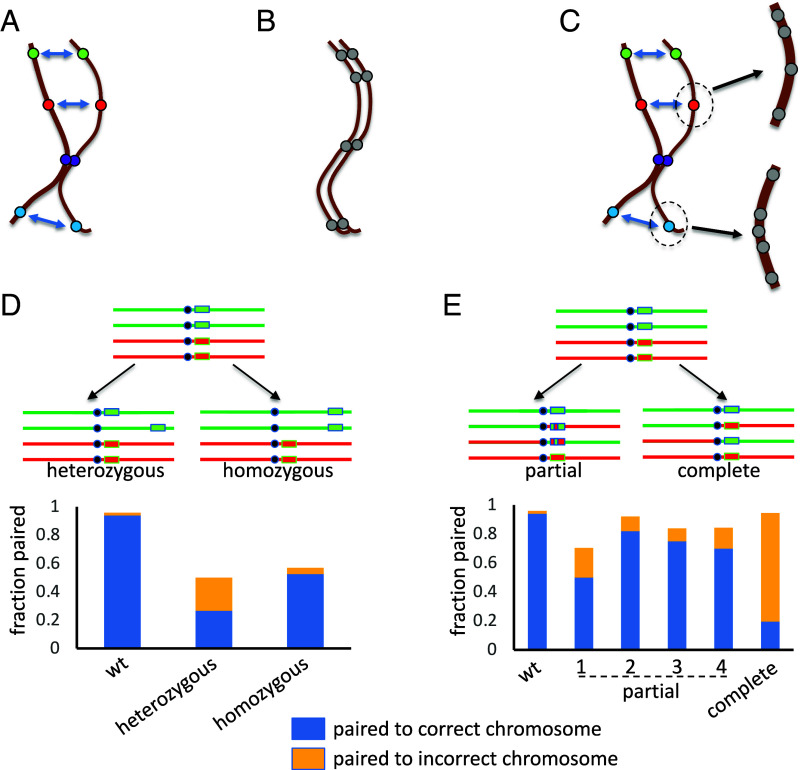
Button models for homolog recognition. (*A*) Specific Button model, in which each pairing button only associates with an identical type found on the homolog, as indicated by the different colors. (*B*) Nonspecific Button Barcode model. (*C*) Barcode Patch model, in which short segments containing nonspecific association domains arranged in distinct bar-code patterns creates larger scale specific buttons, without at any point requiring homolog-specific molecular interactions. (*D*) Simulations of outcomes for a translocation of a button barcode (rectangles) to a distal location on the same chromosome. Graph plots simulation results showing the fraction of nodes paired to the correct (blue) and incorrect (gold) homologs. Simulations were performed using the button barcode from Fig. 5*B* using identical parameters and run times. For the translocation, the patch was shifted distally by 12 nodes in the chain. (*E*) Simulations of outcomes for reciprocal translocations between two different button barcode patches, indicated by red and green rectangles in the diagram. Graph shows outcomes plotted as in panel *D*, for four reciprocal partial translocations, each of which involved swapping a different subset of three nodes between the two chromosomes, such that each set of three nodes spanned one quarter of the total barcode patch which was 12 nodes long. The diagram above the graph shows one of the four possible translocation of a quarter patch. The subsets of nodes swapped in each partial translocation are denoted by the numbers 1 to 4, as well as the outcome for a full translocation of the entire patch between the two chromosomes. The complete translocation was implemented by swapping the entire button barcode patch between two chromosomes. As with panel *D*, the simulation used the optimal barcode pair from Fig. 5*B*.

We have posed our nonspecific button barcode model as being fundamentally different from a specific button model (Fig. 8 *A* and *B*), but this distinction may not actually be so clear. We have shown in our simulations that while barcodes spanning an entire arm can achieve specificity, we also find that even short barcodes involving just a few nonspecific buttons can also pair selectively (Fig. 5*B*). This suggests a variant hybrid model (Fig. 8*C*), in which short barcode segments of nonspecific buttons can selectively associate with each other based on the pattern of spacing between their button elements, but then these barcode segments would serve as selective buttons for overall chromosome pairing.

Another type of variant model we considered was one in which the buttons and gaps between the buttons are swapped, so that buttons become nonpairing nodes, and gaps between buttons become long tracts of pairing sites that would effectively act as a single large site, with discrimination arising from the energetic difference between full vs. partial occupancy of the possible sites in the tract. We simulated this variant for both the five-button codes from Fig. 5*B* and for the 2-of-5 industrial barcode from Fig. 6, with results given in *SI Appendix*, Fig. S2. We find that when buttons and gaps are interchanged, the resulting barcodes still give robust homolog discrimination in most cases, emphasizing that what matters most is that different homologs have different distributions of pairing sites, regardless of whether a code is viewed as consisting of where the pairing sites are, or the gaps between them.

### Effect of Chromosome Translocations on Button Barcode Mediated Pairing.

The model presented here predicts that, depending on the density of buttons, large-scale chromosome rearrangements could potentially disrupt homolog recognition, either by disrupting a barcode if the rearrangement took place within the barcode, or by creating a mismatch in nuclear location due to the Rabl orientation. For example, a translocation would delete part of one bar code and add it to a different bar code on another chromosome. A deletion would alter the number of buttons or the spacing between them while a duplication would add buttons or increase the spacing. Indeed, a model based on a barcode of nonspecific buttons should be much more sensitive to the effects of chromosome rearrangements than a model based on specific buttons, which would retain their specificity if moved to another chromosome. Thus, to distinguish a button barcode model from a specific button model, one key class of experiments might seem to be analysis of the effect of chromosome rearrangements on pairing. In fact, there are two types of experiments involving translocations: testing whether large rearrangements disrupt pairing of homologs, and testing whether translocation of small regions preserves pairing between the translocated region and its original homolog rather than the homolog of the chromosome into which it was translocated. In *Drosophila*, the influence of chromosome rearrangements on somatic pairing has been inferred from the effect of translocations on transvection (30, 31, 59, 60), but these studies are complicated by the fact that chromosome rearrangements might also disrupt the position of sequence elements required for transvection rather than pairing per se.

Direct imaging of pairing in translocation strains is not subject to such concerns. A disruption of homolog pairing at one specific locus, the histone locus on chromosome arm 2L, was directly observed by FISH for the ltx13 translocation in *Drosophila*, which moves most of the left arm of chromosome 2 onto the end of the right arm of chromosome 3. Somatic pairing of the histone locus on 2L is almost completely lost when this translocation is heterozygous, but is mostly restored if the translocation is homozygous (20). In the context of our present model, the effect of these large translocations could potentially be explained by the fact that the large translocations move loci to different positions along the Rabl axis, resulting in two pairing patches being localized in distinct regions of the nucleus where they never get the chance to interact (43, 61). We tested this idea by simulating pairing in which the barcode patch was shifted toward the end of the chromosome. As shown in Fig. 8*D*, this shift in position was enough to greatly disrupt homolog pairing on both chromosomes, without leading to an increase in pairing with the incorrect chromosome. When the shift was present on both copies of the homolog, corresponding to a homozygous translocation, the pairing greatly increased, although still not to wild type levels, which we attribute to the influence of position along the Rabl axis on overall pairing efficiency (Fig. 5*A*). This loss of pairing in a heterozygous translocation to a distal region, combined with recovery of pairing in the homozygous translocation, is consistent with previous experimental results with such translocations (20).

An alternative type of rearrangement used to explore pairing determinants is the transfer of a small genomic region elsewhere in the genome to ask whether such a translocated region is able to confer pairing to the original chromosome. This approach was taken by Viets et al. (32) who identified a number of regions capable of ectopic pairing when relocated in the genome, all of which were on the order of 100 kb in size. However not every region of this size could confer pairing. In the cases where it did confer pairing, the whole region was required since when smaller subregions were tested, they did not have the ability to pair.

To ask whether the Button Barcode model is consistent with this type of translocation data, we simulated pairing in reciprocal translocations in which equal-sized portions of the five-button barcode pair from Fig. 5*B* were swapped between the two chromosomes. As shown in Fig. 8*E*, small reciprocal translocations within the barcode (involving 4 out of 12 nodes) reduced overall pairing efficiency, but did not cause the chromosome bearing the insertion to pair with the source of the insertion, as indicated by the low fraction of incorrectly paired nodes. In contrast, a completely reciprocal translocation of the whole barcode patch led to a new outcome in which the chromosome receiving the translocation paired predominantly with the homolog of the chromosome from which the translocated barcode was derived. These simulations show that small translocations are not sufficient to confer chromosome identity, while a larger translocation relocating an entire barcode patch can indeed switch the homology preference of a chromosome, all of which is consistent with the experimental results of Viets et al. (32).

## Discussion

### Comparison with Pairing Levels Reported in Other Studies.

In our simplified model, we find that nonspecific associations can result in specific pairing frequencies exceeding 90%. However, our model does not reliably achieve 100% pairing for any parameter values we have tried. Our model is not intended to represent any specific actual species or cell type, but rather to be an abstract model to test the general concept of button barcodes. Nevertheless, the fact that the model cannot achieve 100% pairing raises the question of how this compares with what is seen in actual cells?

Chromosome-wide somatic pairing is most notable in *Drosophila*. A FISH survey of 11 loci on the left arm of chromosome 2 in cycle 14 *Drosophila* embryos found a range of pairing frequencies from 7 to 85%, with most loci in the range of 20 to 30% paired (21). The same study found that later in embryonic development, by 6 h AED, pairing frequencies increased to 20 to 98%, eventually reaching 80 to 100% by day 5 of development. Other FISH analyses in *Drosophila* embryos gave pairing frequencies of 60 to 90% for cycle 14 (20) and 70% in postgastrulation embryos (62). In the *Drosophila* eye, Viets et al. (32) found that pairing loci associated at frequencies in the range of 88 to 94%. Screening studies of specific loci under a range of perturbations gave control levels of pairing in the range 47 to 91% for *Drosophila* embryos (63) and 40 to 80% for *Drosophila* Kc167 cells (64).

In human cells that show pairing of only certain chromosome regions, rather than whole chromosomes like in *Drosophila*, pairing frequencies have been reported to be in a similar range (e.g., ref. 22).

It is thus clear that while our model does not achieve 100% pairing efficiency, neither do actual chromosomes. We conclude that the level of pairing achievable even with a simple model based on nonspecific associations can, at least in principle, produce the necessary level of correct pairing.

### Implications of Chromatin Physics for Homology Recognition.

The ability to achieve specific homolog recognition in the nonspecific button barcode model depends on the mechanical properties of the chromosomes and their arrangement in the nucleus (Fig. 3). Are the physical properties of actual chromosomes in an appropriate regime for this type of model to work?

A key question with respect to persistence length in the model is whether, on the length scale of spacing between pairing sites, the chromosome is better treated as a random walk polymer or an elastic rod. To decide which regime applies in the case of *Drosophila* embryos during the time of somatic homolog pairing, we refer to a prior analysis of nuclear position of loci spanning the left arm of Chromosome 2 in cycle 13 *Drosophila* which showed that position along the chromosome was highly correlated with position along the nuclear axis (43), confirming the presence of a strong Rabl orientation in *Drosophila* embryonic nuclei. Replotting that data by averaging positions for loci in a given segment of the arm (each arm of a *Drosophila* chromosome has 20 cytologically defined segments), and then plotting vertical position vs. genome position for eight segments of the left arm of chromosome 2, we find that vertical position is well fit by a linear function (*SI Appendix*, Fig. S3), suggesting that the interphase chromosome is behaving more like an elastic rod than a random chain, and suggesting that the persistence length should be at least on the order of the size of the nucleus, which fits with the 1 to 3 µ range in which we see effective homolog recognition in our model. Given the importance of chromosome elasticity for enforcing correct pairing in this model, one prediction is that DNA replication, which should decompact chromatin, might be expected to disrupt homolog pairing by reducing the persistence length as the chromatin decompacts and becomes more flexible. In fact, analysis of homolog pairing at different cell cycle stages in *Drosophila* has found that this is the case, such that homolog pairing is substantially reduced in G2 relative to G1 and is not fully restored until after cell division (65).

### Potential Molecular Basis for Nonspecific Buttons.

Whether in the context of somatic homolog pairing, or recombination-independent meiotic pairing, testing the nonspecific button barcode model will require identifying the molecules that create the adhesive function of the buttons, allowing their distribution to be altered to change the barcode with predictable outcomes. The molecules that mediate somatic pairing are not known. One potential candidate that has been proposed for such a role in meiotic chromosomes is cohesin (66), but one could invoke a wide range of possible interactions mediated by proteins or other molecules associated with the chromosomes.

In *C. elegans*, each PC contains short sequence elements that bind zinc finger proteins known as ZIMs. Each PC recruits a single type of ZIM (HIM-8 for the X chromosome, ZIM-3 for chromosome 1, etc.), but in some cases, the same ZIM is recruited to more than one PC (for example ZIM-3 is recruited to both chromosome I and chromosome IV). Artificial arrays of these zinc finger binding sequences can replace the normal requirement for a PC and cause ectopic pairing to nonhomologous chromosomes when translocated into new contexts (67). These PCs seem like specific buttons, in that each PC only associates with the homologous PC. The obvious model is that ZIMs recognize the sequences, and then interactions between the ZIMs drive pairing. But this cannot be the whole story because more than one PC shares the same ZIM. Within each PC, the zinc finger binding sequences occur in clusters separated by long stretches of intervening DNA sequence (67). We propose that the differential spacing of zinc finger binding sequences along each PC provides specificity, even when the same ZIMs are used, via the same barcode patch mechanism described here (Fig. 8*C*).

### Barcode Effects in DSB Mediated Pairing.

The concept underlying the button barcode model is that alignment of buttons with unequal spacing on different chromosomes would create an energetic cost that would disfavor such binding in comparison to binding of buttons between chromosomes that had the same spacing between those buttons. There is no obvious reason why such an energetic discrimination mechanism could not also play a role in conventional DSB-mediated meiotic pairing. The same physical effects in our model that favor pairing of nonspecific buttons having similar spacings along their respective chromosomes, would favor a given pair of DSBs on one chromosome associating with homologous regions spaced similarly on the other chromosome. Even if each DSB was potentially capable of base pairing with several different homeologous sequence stretches elsewhere in the genome, these alternative regions will not in general be spaced the correct distance on the nonhomolog, and thus false associations will be disfavored due to chromosome elasticity. It has been shown that mutation in cohesin leads to increased ectopic pairing in yeast (68). We speculate that this decreased fidelity of homolog recognition might result from a reduction in the chromosome stiffness, such that it becomes more like a random walk, and therefore less able to benefit from the proposed mechanical barcode effect. A mechanical contribution to pairing specificity might be particularly likely in species like *Schizosaccharomyces pombe* or *Tetrahymena*, where the chromosomes are drawn out into long parallel “horse tail” configurations (69, 70). This stretching involves clustering of both telomeres and centromeres (71, 72), analogous to the Rabl configuration seen during somatic homolog pairing in *Drosophila*, such that bending of a chromosome to accommodate recombination between sites at different genomic positions would be disfavored.

## Materials and Methods

Chromosomes were modeled using Brownian Dynamics simulation of a bead-spring chain, in which a subset of beads were designated as pairing buttons and able to associate with any other pairing button. The chains were confined to a spherical nucleus, and subject to random thermal forces. Once a pair of buttons were associated, they were allowed to unpair with a defined probability.

## Supplementary Material

Appendix 01 (PDF)

## Data Availability

There are no data underlying this work.

## References

[1] S. Keeney, C. N. Giroux, N. Kleckner, Meiosis-specific DNA double-strand breaks are catalyzed by Spo11, a member of a widely conserved protein family. Cell **88**, 375–384 (1997). 9039264 10.1016/s0092-8674(00)81876-0

[2] P. J. Romanienko, R. D. Camerini-Otero, The mouse Spo11 gene is required for meiotic chromosome synapsis. Mol. Cell **6**, 975–987 (2000). 10.1016/s1097-2765(00)00097-6 11106738

[3] B. D. McKee, R. Yan, J.-H. Tsai, Meiosis in male Drosophila. Spermatogenesis **2**, 167–184 (2012). 23087836 10.4161/spmg.21800 PMC3469440

[4] T. Rubin , Premeiotic pairing of homologous chromosomes during Drosophila male meiosis. Proc. Natl. Acad. Sci. U.S.A. **119**, e2207660119 (2022). 36375065 10.1073/pnas.2207660119 PMC9704699

[5] K. S. McKim, A. M. Howell, A. M. Rose, The effects of translocations on recombination frequency in Caenorhabditis elegans. Genetics **120**, 987–1001 (1988). 3224815 10.1093/genetics/120.4.987 PMC1203590

[6] O. Rog, A. F. Dernburg, Direct visualization reveals kinetics of meiotic chromosome synapsis. Cell Rep. **10**, 1639–1645 (2015). 25772351 10.1016/j.celrep.2015.02.032 PMC4565782

[7] A. F. Dernburg , Meiotic recombination in C. elegans initiates by a conserved mechanism and is dispensable for homologous chromosome synapsis. Cell **94**, 387–398 (1998). 9708740 10.1016/s0092-8674(00)81481-6

[8] M. Zetka, A. Rose, The genetics of meiosis in Caenorhabditis elegans. Trends Genet. **11**, 27–31 (1995). 7900192 10.1016/s0168-9525(00)88983-0

[9] A. J. MacQueen , Chromosome sites play dual roles to establish homologous synapsis during meiosis in C. elegans. Cell **123**, 1037–1050 (2005). 16360034 10.1016/j.cell.2005.09.034 PMC4435800

[10] H. Scherthan, J. Loidl, T. Schuster, D. Schweizer, Meiotic chromosome condensation and pairing in Saccharomyces cerevisiae studied by chromosome painting. Chromosoma **101**, 590–595 (1992). 1424983 10.1007/BF00360535

[11] B. M. Weiner, N. Kleckner, Chromosome pairing via multiple interstitial interactions before and during meiosis in yeast. Cell **77**, 977–991 (1994). 8020104 10.1016/0092-8674(94)90438-3

[12] S. M. Burgess, N. Kleckner, B. M. Weiner, Somatic pairing of homologs in budding yeast: Existence and modulation. Genes Dev. **13**, 1627–1641 (1999). 10385630 10.1101/gad.13.12.1627 PMC316803

[13] K. A. Boateng, M. A. Bellani, I. V. Gregoretti, F. Pratto, R. D. Camerini-Otero, Homologous pairing preceding SPO11-mediated double-strand breaks in mice. Dev. Cell **24**, 196–205 (2013). 23318132 10.1016/j.devcel.2012.12.002 PMC3562373

[14] C. Grey, B. de Massy, Chromosome organization in early meiotic prophase. Front. Cell Dev. Biol. **9**, 688878 (2021). 34150782 10.3389/fcell.2021.688878 PMC8209517

[15] M. Solé , Time to match; when do homologous chromosomes become closer? Chromosoma **131**, 193–205 (2022). 35960388 10.1007/s00412-022-00777-0 PMC9674740

[16] T. A. C. Newman , Diffusion and distal linkages govern interchromosomal dynamics during meiotic prophase. Proc. Natl. Acad. Sci. U.S.A. **119**, e2115883119 (2022). 35302885 10.1073/pnas.2115883119 PMC8944930

[17] J. Loidl, F. Klein, H. Scherthan, Homologous pairing is reduced but not abolished in asynaptic mutants of yeast. J. Cell Biol. **125**, 1191–1200 (1994). 8207053 10.1083/jcb.125.6.1191 PMC2290927

[18] N. M. Stevens, A study of the germ cells of certain diptera, with reference to the heterochromosomes and the phenomena of synapsis. J. Exp. Zool. **5**, 359–374 (1908).

[19] C. W. Metz, Chromosome studies on the Diptera. II. The paired association of chromosomes in the Diptera, and its significance. J. Exp. Zool. **21**, 213–279 (1916).

[20] Y. Hiraoka , The onset of homologous chromosome pairing during Drosophila melanogaster embryogenesis. J. Cell Biol. **120**, 591–600 (1993). 8425892 10.1083/jcb.120.3.591 PMC2119536

[21] J. C. Fung, W. F. Marshall, A. Dernburg, D. A. Agard, J. W. Sedat, Homologous chromosome pairing in Drosophila melanogaster proceeds through multiple independent initiations. J. Cell Biol. **141**, 5–20 (1998). 9531544 10.1083/jcb.141.1.5 PMC2132734

[22] E. P. Arnoldus, A. C. Peters, G. T. Bots, A. K. Raap, M. van der Ploeg, Somatic pairing of chromosome 1 centromeres in interphase nuclei of human cerebellum. Hum. Genet. **83**, 231–234 (1989). 2793166 10.1007/BF00285162

[23] M. S. Apte, V. H. Meller, Homologue pairing in flies and mammals: Gene regulation when two are involved. Genet. Res. Int. **2012**, 430587 (2012). 22567388 10.1155/2012/430587 PMC3335585

[24] E. F. Joyce, N. Apostolopoulos, B. J. Beliveau, C. Wu, Germline progenitors escape the widespread phenomenon of homolog pairing during Drosophila development. PLoS Genet. **9**, e1004013 (2013). 24385920 10.1371/journal.pgen.1004013 PMC3868550

[25] A. Lorenz, J. Fuchs, R. Bürger, J. Loidl, Chromosome pairing does not contribute to nuclear architecture in vegetative yeast cells. Eukaryot. Cell **2**, 856–866 (2003). 14555468 10.1128/EC.2.5.856-866.2003 PMC219365

[26] D. Zickler, N. Kleckner, Meiotic chromosomes: Integrating structure and function. Annu. Rev. Genet. **33**, 603–754 (1999). 10690419 10.1146/annurev.genet.33.1.603

[27] T. Fukaya, M. Levine, Transvection. Curr. Biol. **27**, R1047–R1049 (2017). 29017034 10.1016/j.cub.2017.08.001 PMC6092964

[28] E. F. Joyce, J. Erceg, C.-T. Wu, Pairing and anti-pairing: A balancing act in the diploid genome. Curr. Opin. Genet. Dev. **37**, 119–128 (2016). 27065367 10.1016/j.gde.2016.03.002 PMC4939289

[29] J. AlHaj Abed , Highly structured homolog pairing reflects functional organization of the Drosophila genome. Nat. Commun. **10**, 4485 (2019). 31582763 10.1038/s41467-019-12208-3 PMC6776532

[30] E. B. Lewis The theory and application of a new method of detecting chromosomal rearrangements in Drosophila Melanogaster Am. Nat. **88**, 225-239 (1954).

[31] S. A. Ou , Effects of chromosomal rearrangements on transvection at the yellow gene of Drosophila melanogaster. Genetics **183**, 483–496 (2009). 19667134 10.1534/genetics.109.106559 PMC2766311

[32] K. Viets , Characterization of button loci that promote homologous chromosome pairing and cell-type-specific interchromosomal gene regulation. Dev. Cell **51**, 341–356.e7 (2019). 31607649 10.1016/j.devcel.2019.09.007 PMC6934266

[33] M. B. Child VI , Live imaging and biophysical modeling support a button-based mechanism of somatic homolog pairing in Drosophila. Elife **10**, e64412 (2021). 34100718 10.7554/eLife.64412 PMC8294847

[34] M. Nicodemi, B. Panning, A. Prisco, The colocalization transition of homologous chromosomes at meiosis. Phys. Rev. E Stat. Nonlin. Soft Matter Phys. **77**, 061913 (2008). 18643306 10.1103/PhysRevE.77.061913

[35] R. C. Palmer, The Bar Code Book (Helmers Publishing Inc., 1995).

[36] D. Allais, Bar Code Symbology (Intermec Corp. Report, 1984).

[37] L. Ehrlich, C. Münkel, G. Chirico, J. Langowski, A Brownian dynamics model for the chromatin fiber. Comput. Appl. Biosci. **13**, 271–279 (1997). 9183532 10.1093/bioinformatics/13.3.271

[38] J. F. Marko, E. D. Siggia, Polymer models of meiotic and mitotic chromosomes. Mol. Biol. Cell **8**, 2217–2231 (1997). 9362064 10.1091/mbc.8.11.2217 PMC25703

[39] K. Bystricky, P. Heun, L. Gehlen, J. Langowski, S. M. Gasser, Long-range compaction and flexibility of interphase chromatin in budding yeast analyzed by high-resolution imaging techniques. Proc. Natl. Acad. Sci. U.S.A. **101**, 16495–16500 (2004). 15545610 10.1073/pnas.0402766101 PMC534505

[40] C. A. Penfold, P. E. Brown, N. D. Lawrence, A. S. H. Goldman, Modeling meiotic chromosomes indicates a size dependent contribution of telomere clustering and chromosome rigidity to homologue juxtaposition. PLoS Comput. Biol. **8**, e1002496 (2012). 22570605 10.1371/journal.pcbi.1002496 PMC3342934

[41] W. F. Marshall, J. C. Fung, Modeling meiotic chromosome pairing: Nuclear envelope attachment, telomere-led active random motion, and anomalous diffusion. Phys. Biol. **13**, 026003 (2016). 27046097 10.1088/1478-3975/13/2/026003 PMC5120002

[42] W. F. Marshall, J. C. Fung, Modeling meiotic chromosome pairing: A tug of war between telomere forces and a pairing-based Brownian ratchet leads to increased pairing fidelity. Phys. Biol. **16**, 046005 (2019). 30943453 10.1088/1478-3975/ab15a7 PMC6581521

[43] W. F. Marshall, A. F. Dernburg, B. Harmon, D. A. Agard, J. W. Sedat, Specific interactions of chromatin with the nuclear envelope: Positional determination within the nucleus in Drosophila melanogaster. Mol. Biol. Cell **7**, 825–842 (1996). 8744953 10.1091/mbc.7.5.825 PMC275932

[44] A. I. Grosberg, A. R. Khokhlov, Statistical Physics of Macromolecules (AIP Press, 1994).

[45] A. Garai, S. Saurabh, Y. Lansac, P. K. Maiti, DNA elasticity from short DNA to nucleosomal DNA. J. Phys. Chem. B **119**, 11146–11156 (2015). 26134918 10.1021/acs.jpcb.5b03006

[46] W. F. Marshall, J. F. Marko, D. A. Agard, J. W. Sedat, Chromosome elasticity and mitotic polar ejection force measured in living Drosophila embryos by four-dimensional microscopy-based motion analysis. Curr. Biol. **11**, 569–578 (2001). 11369201 10.1016/s0960-9822(01)00180-4

[47] M. G. Lowenstein, T. D. Goddard, J. W. Sedat, Long-range interphase chromosome organization in Drosophila: A study using color barcoded fluorescence in situ hybridization and structural clustering analysis. Mol. Biol. Cell **15**, 5678–5692 (2004). 15371546 10.1091/mbc.E04-04-0289 PMC532046

[48] J. R. Swedlow, T. Hirano, The making of the mitotic chromosome: Modern insights into classical questions. Mol. Cell **11**, 557–569 (2003). 12667441 10.1016/s1097-2765(03)00103-5

[49] N. Kleckner, B. M. Weiner, Potential advantages of unstable interactions for pairing of chromosomes in meiotic, somatic, and premeiotic cells. Cold Spring Harb. Symp. Quant. Biol. **58**, 553–565 (1993). 7956070 10.1101/sqb.1993.058.01.062

[50] D. E. Comings, Arrangement of chromatin in the nucleus. Hum. Genet. **53**, 131–143 (1980). 6987157 10.1007/BF00273484

[51] J. Brickner, Genetic and epigenetic control of the spatial organization of the genome. Mol. Biol. Cell **28**, 364–369 (2017). 28137949 10.1091/mbc.E16-03-0149 PMC5341720

[52] C. Vourc'h, D. Taruscio, A. L. Boyle, D. C. Ward, Cell cycle-dependent distribution of telomeres, centromeres, and chromosome-specific subsatellite domains in the interphase nucleus of mouse lymphocytes. Exp. Cell Res. **205**, 142–151 (1993). 8453988 10.1006/excr.1993.1068

[53] J. A. Croft , Differences in the localization and morphology of chromosomes in the human nucleus. J. Cell Biol. **145**, 1119–1131 (1999). 10366586 10.1083/jcb.145.6.1119 PMC2133153

[54] M. Cremer , Non-random radial higher-order chromatin arrangements in nuclei of diploid human cells. Chromosome Res. **9**, 541–567 (2001). 11721953 10.1023/a:1012495201697

[55] C. Carvalho , Chromosomal G-dark bands determine the spatial organization of centromeric heterochromatin in the nucleus. Mol. Biol. Cell **12**, 3563–3572 (2001). 11694589 10.1091/mbc.12.11.3563 PMC60276

[56] A. F. Dernburg , Perturbation of nuclear architecture by long-distance chromosome interactions. Cell **85**, 745–759 (1996). 8646782 10.1016/s0092-8674(00)81240-4

[57] T. Pavlidis, J. Swartz, Y. P. Wang, Fundamentals of bar code information theory. IEEE Comput. **23**, 74–86 (1990).

[58] C. K. Harmon, Reading between the Lines: An Introduction to Bar Code Technology (Helmers Pub, ed. 4, 1989).

[59] S. M. Smolik-Utlaut, W. M. Gelbart, The effects of chromosomal rearrangements on the zeste-white interaction in Drosophila melanogaster. Genetics **116**, 285–298 (1987). 3111938 10.1093/genetics/116.2.285 PMC1203139

[60] I. W. Duncan, Transvection effects in Drosophila. Annu. Rev. Genet. **36**, 521–556 (2002). 12429702 10.1146/annurev.genet.36.060402.100441

[61] J. Erceg , The genome-wide multi-layered architecture of chromosome pairing in early Drosophila embryos. Nat. Commun. **10**, 4486 (2019). 31582744 10.1038/s41467-019-12211-8 PMC6776651

[62] M. J. Gemkow, P. J. Verveer, D. J. Arndt-Jovin, Homologous association of the Bithorax-Complex during embryogenesis: Consequences for transvection in Drosophila melanogaster. Development **125**, 4541–4552 (1998). 9778512 10.1242/dev.125.22.4541

[63] J. R. Bateman, C.-t. Wu, A genomewide survey argues that every zygotic gene product is dispensable for the initiation of somatic homolog pairing in Drosophila. Genetics **180**, 1329–1342 (2008). 18791221 10.1534/genetics.108.094862 PMC2581938

[64] M. Puerto , Somatic chromosome pairing has a determinant impact on 3D chromatin organization. bioRxiv [Preprint]. 10.1101/2023.03.29.534693 (2023).

[65] A. K. Csink, S. Henikoff, Large-scale chromosomal movements during interphase progression in Drosophila. J. Cell Biol. **143**, 13–22 (1998). 9763417 10.1083/jcb.143.1.13 PMC2132807

[66] K.-I. Ishiguro , Meiosis-specific cohesin mediates homolog recognition in mouse spermatocytes. Genes Dev. **28**, 594–607 (2014). 24589552 10.1101/gad.237313.113 PMC3967048

[67] C. M. Phillips , Identification of chromosome sequence motifs that mediate meiotic pairing and synapsis in C. elegans. Nat. Cell Biol. **11**, 934–942 (2009). 19620970 10.1038/ncb1904 PMC4001799

[68] D. Y. Lui, C. K. Cahoon, S. M. Burgess, Multiple opposing constraints govern chromosome interactions during meiosis. PLoS Genet. **9**, e1003197 (2013). 23341780 10.1371/journal.pgen.1003197 PMC3547833

[69] Y. Chikashige , Telomere-led premeiotic chromosome movement in fission yeast. Science **264**, 270–273 (1994). 8146661 10.1126/science.8146661

[70] J. Loidl, H. Scherthan, Organization and pairing of meiotic chromosomes in the ciliate Tetrahymena thermophila. J. Cell Sci. **117**, 5791–5801 (2004). 15522890 10.1242/jcs.01504

[71] J. Loidl, A. Lukaszewicz, R. A. Howard-Till, T. Koestler, The Tetrahymena meiotic chromosome bouquet is organized by centromeres and promotes interhomolog recombination. J. Cell Sci. **125**, 5873–5880 (2012). 22976299 10.1242/jcs.112664

[72] M. Tian, C. Agreiter, J. Loidl, Spatial constraints on chromosomes are instrumental to meiotic pairing. J. Cell Sci. **133**, jcs253724 (2020). 33172984 10.1242/jcs.253724 PMC7725606

