# Supplementary material for: Modeling homologous chromosome recognition via nonspecific interactions
Source: Proc Natl Acad Sci U S A. 2024 May 9;121(20):e2317373121. doi: 10.1073/pnas.2317373121 (PMC11098084; doi:10.1073/pnas.2317373121)
Supplement: Supplementary file 1 — Appendix 01 (PDF) [file pnas.2317373121.sapp.pdf]

**Supporting Information for**
Paste manuscript title here.

Paste the full author list Wallace F. Marshall, Jennifer C. Fung

Paste corresponding author name Wallace Marshall and Jennifer C. Fung
Email: Jennifer.Fung@ucsf.edu

**This PDF file includes:**

Supporting text
Figures S1 to S3
SI References

## Supporting Information Text

## Materials and Methods

### *Coarse grained polymer model for chromosome dynamics*

The model used is based on a previous coarse-grained Langevin model developed for meiotic chromosome movement (1, 2). In this model, each chromosome is represented as a string of beads joined by springs. Each bead (node) is subject to a random thermal (Langevin) force, a frictional force proportional to velocity, and additional force terms that describe the physical properties of the chromosome and its interaction with the nuclear envelope.

These forces yield the following equation of motion, which is used to update the velocity and position of each node at each time step:

$$\zeta\vec{v} = -k_s(\|\hat{x}_i - \hat{x}_{i-1}\| - L_{eq})\hat{u}_i + k_s(\|\hat{x}_i - \hat{x}_{i+1}\| - L_{eq})\hat{u}_{i+1} + \sigma\hat{n}_r$$

where $x_i$ and $v_i$ are position and velocity vectors corresponding to node i on the chain, $\zeta$ is the friction coefficient, $k_s$ and $L_{eq}$ are the spring constant and equilibrium length of the links, $\sigma$ is the average magnitude of the Langevin (thermal) random force, and $n_r$ is a randomly oriented unit vector generated for each node at every time point, and $u_i$ represents a unit vector directed from node i-1 to node i. We represent a worm like chain using the Kratky-Porod model in which a force is generated proportional to the bending angle between two successive links with a proportionality constant $k_{bend}$.

The Langevin random force actually represents the average net resultant of all random collisions with thermally excited solvent molecules during a single time-step. The magnitude of the Langevin random force is assumed to be constant at every time step, so $\sigma$ is constant. At each time point, a new orientation is chosen for each of the random unit vectors $n_r$ is chosen from a uniform distribution of angles in spherical coordinates.

In this model, we employ a Rouse "phantom polymer" model in which the chromatin chains are able to freely pass through each other. This assumption is supported by polymer scaling arguments based on the relative density of topoisomerase strand-passage sites compared to the density of chromatin interlocks , which indicate that any interlocks that form would be rapidly resolved by topoisomerase activity (3), a behavior that is also supported by experimental measurements of topoisomerase ability to convert an entangled DNA melt into a viscous fluid (4). While this assumption may not be appropriate for meiotic chromosomes, which consist of four strands each and in many cases also contain protein-based axial elements, which would prevent strand passage and therefore require a model that can capture entanglement effects (5), in the case of somatic homolog pairing, these concerns do not apply and so the Rouse assumption is justified. During motion, the chromosomes are confined inside a spherical nuclear envelope by a repulsive force applied to any node moving outside the spherical shell in a direction normal to the surface with a spring constant k_nuc as previously described (1).

To simulate chromosome dynamics during pairing, a chain is initialized to a random configuration using a lattice model, and then relaxed according to the Langevin equation and nuclear constraints, as previously described (1). After the initial relaxation period is over, subsequent timesteps consist of the following steps. First, a test is made to see if any of the currently paired nodes will become unpaired. Then, the displacements calculated for each node in the previous iteration are applied to all nodes. After the node positions are updated, a test is made to see if any nodes in the pairing list are within a capture radius of each other, and if so, they are assigned to the paired state with probability p_pair. Finally, new velocities are calculated for every node, taking into account the current set of node positions, according to the equation above.

### *Modeling nonspecific interactions*

For each simulation, a list of nodes capable of pairing (the "buttons") is specified. As the bead-spring chains undergo their random movement, whenever two loci on the button lists for any of the chromosomes move to with a defined capture radius of each other, they are set to a paired state. This pairing process does not discriminate between buttons on different chromosomes - any button is allowed to pair with any other button, including on the same chromosome, its correct homolog, or either of the other chromosomes. When a button is set to the paired state, the identifier of the button to which it is paired is stored, and from then on the motion of the two nodes will subsequently be forced to coincide (details for enforcing this correlated motion are given in previous work, Marshall and Fung 2016 (1)). Once a pair of buttons are set to the paired state and assigned to each other, they are no longer included in subsequent pairing tests.

At subsequent time-steps, unpairing of paired loci occurs with a fixed probability given by the unpairing probability p_unpair. In this model, the value of p_unpair is the same for all pairs of buttons, so that discrimination between correct and incorrect associations can only be achieved by higher order structural or mechanical influences, and not on the local interaction kinetics of the buttons themselves. If two nodes become unpaired, they are set to the unpaired state but not moved apart. The unpairing test is done at the start of each iteration, and whether the nodes will rapidly re-pair depends on whether their random motions during the timestep take them further apart than r_capture during the iteration.

***Modeling the Rabl configuration***

In many cells, chromosomes are arranged in a Rabl configuration, in which telomeres cluster together at one end of the nucleus and centromeres at the other (6). This is conspicuously true in *Drosophil*a embryos at the time of establishment of somatic pairing (7, 8). The Rabl configuration arises as a remnant of chromosome organization during the preceding anaphase, in which the centromeres move together to each pole of the spindle. In addition, chromosomes are often associated with the nuclear envelope (9), with specific chromosome regions, such as telomeres or heterochromatic repeats, non-randomly located in the nuclear periphery. Again, this is particularly evident in *Drosophila* embryos (7). We represent the Rabl configuration by confining the centromeres, defined as the midpoint of each chromosome polymer, to a restricted region on the nuclear surface by applying a restoring force, proportional to the distance to the boundary of the region, to any centromeres that move outside of the region. The size of this region is given by the parameter confinement radius. Within the region, the centromeres are subject to the standard Langevin random force of the model, but they are prevented from leaving the region of constraint.

***Parameter choice:***

The model presented here is intended to ask whether non-specific interactions can, in principle, provide specificity of homologous association. It is not intended to accurately represent the details of any specific biological system. Here we present the choices for parameter values used in this model, in which we have tried to assume simple values that are consistent to a first order with biologically measured values, when such are known. For each parameter in the following discussion, we list the default value used in our simulations along with an estimate of what this value corresponds to in real units.

Within the model, we need to define units of length, time, and force. We begin by determining units that make our assumed values of link length, time step, and Langevin force have reasonable values. We then use these units to convert our other parameters into real units, which we can then compare with estimates in real systems.

**Link length ($L_{eq}$)      1      200 nm**

To estimate the link length in the bead-spring chain, we note that each arm of the chromosome has 25 links. We previously measured the position of multiple loci on one arm of *Chromosome* 2 in *Drosophila* (during cycle 13) and found that the chromosome spans a linear distance of 4 microns (7), which would correspond to 160 nm per link. For our simplified model,

we assume a slightly greater link length of 200 nm. Given that a *Drosophila* chromosome is approximately 50 Mb in size, each 200 nm link would correspond to approximately 1Mb of DNA. Sun et al. (10) has reported that the size range of topological associated domains (TADs) in *Drosophila* embryos is in the range of 0.3-1 Mb, roughly consistent with our model assumptions.

**Time Step 1 10 ms**

To estimate what length of time each step of the simulation corresponds to, we start by noting that the total simulation is carried out for 300,000 steps. The duration of cycle 14 in *Drosophila*, when somatic pairing occurs, is approximately 1 hour. 300,000 steps per hour corresponds to 12 ms per step. We simplify here by setting the time step to 10 ms.

**Langevin force 0.15 0.3 pN**

To estimate the Langevin force used in our simulation, we start with the measured value of D=2.0x10$^{-11}$ cm$^2$/sec for the diffusion coefficient of interphase chromatin measured experimentally in *Drosophila* embryos (Marshall 1997), which is equal to D=2000 nm$^2$/sec. We then use the relation δ=sqrt(6ΔtD), to get the root mean-squared (rms) displacement during a single 10 ms time step:

δ= sqrt(6 * 10 ms * 2000 nm^2/sec * 1sec/1000 ms) = 10.95 nm

which we approximate as 11 nm. We make the assumption that during a single time step, this displacement is driven by continuous application of thermal energy of magnitude $k_BT$, which we take as approximately 4 pN nm at 30C.

Thus, the Langevin force is $k_BT$/δ = 4 pNnm / 11 nm = 0.26 pN, which we approximate as 0.3 pN.

**Unit Conversion Factors**

With these three values estimated, we can compare the parameter values used in the simulation to the physical values they correspond to, and thereby compute the units of all other parameters in the simulation. For the force units, we divide the actual Langevin force (0.3 pN) by the Langevin force parameter used in the simulation (0.15 force units). The unit conversion factors are then as follows:

1 time unit = 10 ms
1 distance unit = 200 nm
1 force unit = 2 pN

**Friction coefficient (ζ) 1.0 3x10$^{-4}$ pN s /nm**

To derive the frictional coefficient ζ, we note that a node of the chain moves by δ=11 nm in a single timestep corresponding to 0.01 s, creating an apparent velocity on the order of 1000 nm/s. This motion is assumed to be driven by the Langevin force of 0.3 pN acting continuously during the time step, driving the node at a constant velocity. From the relation ζ=σ/v we obtain a value for ζ of 0.3 pN / (1000 nm/s) = 3x10$^{-4}$ pN s/nm which is equivalent to 3x10$^{-7}$ Kg/s.

To see if this is reasonable, we can calculate the viscosity corresponding to this frictional coefficient assuming Stoke's law for a perfect sphere, such that η= ζ/6πR. Assuming the de Gennes model of spherical globules whose diameter matches the link length (11), we take R=100 nm = 10$^{-7}$ m, which yields a value for viscosity of (3x10$^{-7}$ Kg/s)/(6*3.14*10$^{-7}$ m) = 0.08 Kg/ms = 4 cP.

We thus estimate a viscosity that is on the order of four times more viscous than water (viscosity 1 cP), consistent with prior studies of the viscosity for the fluid phase of nucleoplasm (12–14).

**Spring constant ($k_s$) 0.25 0.0025 pN/nm**

The parameter $k_s$ is a spring constant describing the force needed to extend a single link in the chain by 1 length unit. To put this into real units we take the product 0.25 force units / length unit * 2pN/force unit / (200 nm/length unit) = 0.0025 pN/nm.

**Torsion spring constant (k_bend) 0-0.4 0 - 0.8 pN**

Our wormlike chain model imposes a force component to oppose bending of the chain. We chose the range of values for the bending parameter k_bend empirically to generate a relevant range of persistence lengths, via simulations with a large (100 length units) nuclear radius to avoid interactions with the NE. From these simulations, we observed a linear relation, with Lp = 40*kbend.

Our minimum value for k_bend is zero, in which case no penalty is imposed for bending the chain, and the model becomes a freely jointed chain. In this case, the persistence length Lp is 100 nm. Our maximum value of kbend is 0.4 force units, which corresponds to 0.8 pN for k_bend.

**Nuclear Envelope Spring Constant (k_nuc) 0.35 3.5 pN per micron**

We represent the nuclear boundary as an elastic shell such that any node of a chromosome chain that moves beyond the specified nuclear radius experiences an inwardly directed radial force proportional to the distance it has moved past the radius. Loci located at or within the nuclear radius do not experience any such force. Representation of the NE as a linear spring is consistent with prior mechanical measurements of nuclear deformation (15–18). Measurements using AFM and optical tweezers to probe the apparent spring constant of the nuclear surface in Xenopus oocytes (17) and mouse embryonic fibroblasts (19) have yielded values in the range 5-20 pN/micron.

**Nuclear radius (nuc_radius) 15.0 3 microns**

The nucleus radius of 15 length units corresponds to 3 microns, comparable to the size scale of cycle 13 nuclei. During cycle 14, nuclei become narrower but lose their spherical shape and become elongated. Here, for simplicity, we assume a spherical nuclear shape.

**Unpairing probability (P_unpair) 0.2-0.8 50 ms**

P_unpair 0.2 means that the average time two loci spend in the paired state, after pairing would be 5 time units, which is just 50 ms. This is orders of magnitude shorter than the reported times that paired loci spend in the paired state (20, 21). However, the avidity effect means that most of the time, a pair of buttons that became unpaired would re-pair so rapidly that the event would not have been detected in live imaging. Unless the loci paired were the only paired loci on a chromosome, they would be highly likely to re-associate rather than drift apart. Standard methods for visualizing chromosome unpairing, based on the presence of one versus two distinct spots in fluorescence imaging, would not in general be able to detect short transient unpairing events. As a technical note, the check for unpairing is done at the start of each simulation time-step, after which the nodes are displaced according to the forces previously calculated for each node. If the displacements are small, the nodes may still be within the capture radius of each other, and thus be able to re-pair with probability p_pair before the forces are calculated for the next iteration.

**Pairing probability (P_pair) 1.0**

We assume that whenever two buttons come within the capture radius of each other, they will pair with a defined probability. As noted above, this pairing is reversible. Once two nodes are paired, their subsequent motion is forced to be equal, with forces on the pair of nodes obtained as the resultant of the forces applied to each of the two nodes in the pair.

**Capture distance (Dist_capture) 0.5 100 nm**

The capture distance was chosen to allow sites to pair when the polymer globules represented by the links are overlapping by the radius of the globule. Under the standard De Gennes globule model, the diameter of a globule equals the link length of a bead-spring representation, so we take half a link length as the capture radius.

**Confinement radius 2.0 400 nm**

This parameter reflects the size of the region of confinement of the centromeres which is used to model the Rabl configuration. In *Drosophila* embryos, centric heterochromatin of all the chromosomes is localized in "chromocenters" that are on the order of 1 micron in diameter (22, 23). Unless noted otherwise, all of the simulations reported here used a small confinement radius of 2.0 length units corresponding to 400 nm. However, in simulations adjusting the confinement radius (**Figure 3D**), no real effect was seen on the Rabl correlation coefficient, our metric for the strength of Rabl alignment of the chromosomes along the z axis, until the radius was greater than 10 length units, corresponding to 4 microns. Thus, the size of the confinement region required to maintain an effective Rabl orientation is well within the range of sizes consistent with the size of *Drosophila* chromocenters.

**n_steps 300000 50 min**

All simulations were run for a total of 300,000 timesteps. Given that each step represents 10 ms, this is equivalent to 50 minutes of real time. The entire duration of cycle 14 in the *Drosophila* embryo is roughly 1 hour, so this is comparable to the time normally available to achieve a high level of somatic pairing.

**initial_relaxation 20000 200 s**

At the start of the simulation, each chromosome is initialized by picking a random point inside the nucleus as the first node, and then adding additional nodes according to a random walk, with the choice of node positions limited to points inside the nucleus. Once this initial configuration is generated, the simulation is run for 20000 iterations, without any pairing, in order to allow the chromosome to relax into a configuration that is consistent with the simulated forces. This number of steps was chosen based on simulations in which the nuclear envelope repulsion force was turned off and the simulation run until the end-to-end distance distribution had reached a steady state (1). After the initial relaxation phase, the simulation continues to run but now pairing is turned on.

### *Energetics of homolog discrimination*

The button barcode model described here relies on mechanical energy to discriminate correct from incorrect pairing. In order to get an idea of the range of energies involved, we calculated the apparent difference in free energy of unpairing for correct versus incorrect pairing (equal versus unequal spacing between buttons) from the simulation results as follows. First, in each simulation we record the fraction of time that a pair of buttons spend correctly paired with their homologs. When both pairs of buttons have the same spacing, we refer to this probability as $P_{matched}$. If the two pairs of buttons have different spacings, we refer to this probability as $P_{mismatched}$. These probabilities can be related to the free energy of pairing the two buttons, using Maxwell-Boltzmann statistics. We are primarily interested not in the energy of association for the pairs of buttons, but in the difference in the energy of association for pairs of buttons with matched versus unmatched spacing between them. Assuming the chromosomes have the same energy when the buttons are unpaired for either spacing, we calculate the difference in free energy change between matched and mismatched button pairs using the following equation derived from the Maxwell-Boltzmann equation:

$$\Delta\Delta G = k_B T^* \{ \ln(P_{matched}) - \ln(P_{mismatched}) \} \qquad \text{(S1)}$$

$\Delta\Delta G$ is the energetic cost of pairing buttons with different spacing relative to pairing buttons with identical spacing, and is a measure of the energetic barrier to incorrect pairing. Using this formula, we can convert the fraction of time spent in the fully paired state, obtained by simulation, into a measure of the free energy difference between full pairing of the correct versus incorrect chromosomes.

In **Supplemental Figure S1A**, $\Delta\Delta G$ is plotted for different levels of mismatch between button spacings, for four different values of correct spacing. Two general trends are apparent in this plot.

First, the range of energies is several times $k_B T$, consistent with the idea that pairing in this model is achieved by relatively weak interactions. Second, as the level of mismatch between the two pairs of buttons increases, the energetic cost to pairing them is greater. There is, however, substantial scatter of energies for any given mismatch, indicating that the energetic cost depends not only on the mismatch but also on the spacing between buttons.

In order to predict how the energetic cost of a mismatch relates to the spacings between the two pairs of buttons, we modeled the two pairs of buttons as being joined by parallel springs, such that when the buttons are paired, the two springs must have the same end-to-end lengths. We consider the free energy needed to deform a pair of chromosomes such that two buttons separated by a length L1 on one chromosome can associate with two buttons separated by length L2 on another chromosome. We approximate the chromatin in between the two buttons as a Hookean spring, which is appropriate both for elastic rods (which act like springs for small deformations (24)) and also for ideal random polymers, as well as polymers with volume exclusion under small deformations (11). For worm-like chain polymers, Hooke's law applies for sufficiently small length changes, with correction terms of higher order applying as the size of the relative length change increases (25). To develop an approximate model, we will here ignore those higher order correction terms. Since we are interested in the case when the spacing between the buttons is unequal, we will let L1 describe the chromosome on which the buttons are more closely spaced, such that L1 < L2. Considering segments of the chromosome as springs, this means that the spring denoted by L1 has a shorter resting length than the spring denoted by L2. We make the further simplifying assumption that the two springs are maintained in parallel with each other.

In order for the buttons to align, spring 1 must stretch by some amount ΔL1, and spring 2 compress by some amount ΔL2. Because both ends of the two springs are coincident with each other, it must be true that

$$L_2 - L_1 = \Delta L_1 + \Delta L_2 \qquad \text{(S2)}$$

When the buttons are paired with each other, the two springs representing the chromatin in between each pair of buttons will reach equilibrium at some point where the force exerted by stretching the shorter spring is equal and opposite the force exerted by compressing the longer spring. The condition for equilibrium is

$$k_1 \Delta L1 = k_2 \Delta L2 \qquad \text{(S3)}$$

where k1 and k2 are spring constants (k1 = EA/L1 and k2 = EA/L2 in terms of the Young's modulus E and cross-sectional area A). Assuming that E and A are the same for both chromosomes, the force balance equation is just:

$$\Delta L1 / L1 = \Delta L2 / L2 \qquad \text{(S4)}$$

Which, upon substitution into (1), yields

$$L2 - L1 = (\Delta L1/L1)*(L1 + L2) \qquad \text{(S5)}$$

Such that

$$\Delta L1 / L1 = \Delta L1 / L1 = (L2 - L1)/(L1 + L2) \qquad \text{(S6)}$$

The total free energy difference between the two-spring system when the spring are connected (buttons paired) and when the springs are separate at their own rest lengths (buttons not paired) is given by the sum of the elastic energies in the two springs, which from Hooke's law can be seen to be

$$\Delta\Delta G = EA\left(\frac{L2-L1}{L1+L2}\right)^2 \qquad \text{(S7)}$$

It is evident that if the two resting lengths are equal (which would be the case for pairing to the correct homolog), the free energy difference is zero. We conclude that the free energy of discrimination between correct versus incorrect paired sets of buttons is quadratic function of (L2-L1)/(L1+L2).

As shown in **Supplemental Figure S1B**, when we re-plot the same results from panel A, but now plotting $\Delta\Delta G$ versus the function of L1 and L2 in equation S7, now all the points collapse onto a straight line (to within error) suggesting that our simplified physical model is usefully depicting the energetics of homology discrimination. In **Supplemental Figure S1C**, we plot $\Delta\Delta G$ for one particular pair of button spacings (L1=3, L2=4) and vary the model parameter $k_s$ which is the spring constant for the springs in our bead-spring polymer model. **Supplemental Figure S1C** shows a roughly linear dependence, consistent with the linear dependence on Youngs modulus. In reality, the effective Young's modulus depends not just on $k_s$ but also $k_{bend}$ for a worm-like chain, which we believe explains why the plot does not go through the origin. All simulations in this paper use a value for $k_s$ of 0.25, which is in the linear regime for this plot.

***Random button code generation***

To generate random button patterns, we used the randperm() function of Matlab to permute a list of n nodes, and then took the first m to be buttons which were then input to the simulation program. In the case of random codes for comparison to the 2 of 5 code, for which there is always a button at both ends of the region in question, we assigned the endpoints of the region to be buttons, and then applied the random permutation scheme to the intervening n-2 nodes.

***Implementing an industrial barcode***

To convert code 2 of 5 (26) into a button pattern spanning a chromosome arm, we assign a button to node 25 (the centromere) and then add additional buttons at spacings given by the following rule: for a narrow bar in 2 of 5, the next button is spaced 2 nodes away. For a wide bar in 2 of 5, the next button is spaced 6 nodes away. This rule preserves the 1:3 ratio of narrow to wide bars used in the industrial code. The resulting button patterns corresponding to the digits in 2 of 5 are as follows, where the binary strings represent the normal way that 2 of 5 is written in text, with 0 corresponding to a narrow bar and 1 to a wide bar (27)

| | | |
|---|---|---|
| 0: | 00110 | 25, 27, 29, 35, 41, 43 |
| 1: | 10001 | 25, 31, 33, 35, 37, 43 |
| 2: | 01001 | 25, 27, 33, 35, 37, 43 |
| 3: | 11000 | 25, 31, 37, 39, 41, 43 |
| 4: | 00101 | 25, 27, 29, 35, 37, 43 |
| 5: | 10100 | 25, 31, 33, 39, 41, 43 |
| 6: | 01100 | 25, 27, 33, 39, 41, 43 |
| 7: | 00011 | 25, 27, 29, 31, 37, 43 |
| 8: | 10010 | 25, 31, 33, 35, 41, 43 |
| 9: | 01010 | 25, 27, 33, 35, 41, 43 |

The simulations in **Figure 6** used code 2 of 5 symbols for 0 and 1.

**Simulation of chromosome translocations**

For simulations of chromosome translocation, we start with the button code from **Figure 5A** in which the first button is located at the centromere (node 25 on a 50 node bead-spring polymer chain), with the buttons on the four chromosomes as follows:

[25, 28, 32, 34, 35] [25, 28, 32, 34, 35] [26, 30, 32, 36, 37] [26, 30, 32, 36, 37]

To simulate a translocation in which one of the button tracts is translocated distally towards the end of the chromosome, we shift the first tract by 12 nodes to create a new barcode. We represent the heterozygous and homozygous translocations thus:

Heterozygous:
[37, 40, 44, 46, 47] [25, 28, 32, 34, 35] [26, 30, 32, 36, 37] [26, 30, 32, 36, 37]

Homozygous:
[37, 40, 44, 46, 47] [37, 40, 44, 46, 47] [26, 30, 32, 36, 37] [26, 30, 32, 36, 37]

To simulate reciprocal translocations we used the following barcodes for the four chromosomes to represent four different partial exchanges each covering one quarter of the barcode patch, and then a full exchange of the whole barcode patch:

Partial exchanges:
[25, 28, 32, 34, 35] [26, 32, 34, 35], [25, 28, 30, 32, 36, 37], [26, 30, 32, 36, 37]
[25, 28, 32, 34, 35] [25, 28, 30, 32, 34, 35], [26, 32, 36, 37], [26, 30, 32, 36, 37]
[25, 28, 32, 34, 35] [25, 28, 32, 35], [26, 30, 32, 34, 36, 37], [26, 30, 32, 36, 37]
[25, 28, 32, 34, 35] [25, 28, 32, 34, 36, 37], [26, 30, 32, 35], [26, 30, 32, 36, 37]

Full exchange:
[25, 28, 32, 34, 35] [26, 30, 32, 36, 37] [25, 28, 32, 34, 35] [26, 30, 32, 36, 37]

**Supplemental Figures**

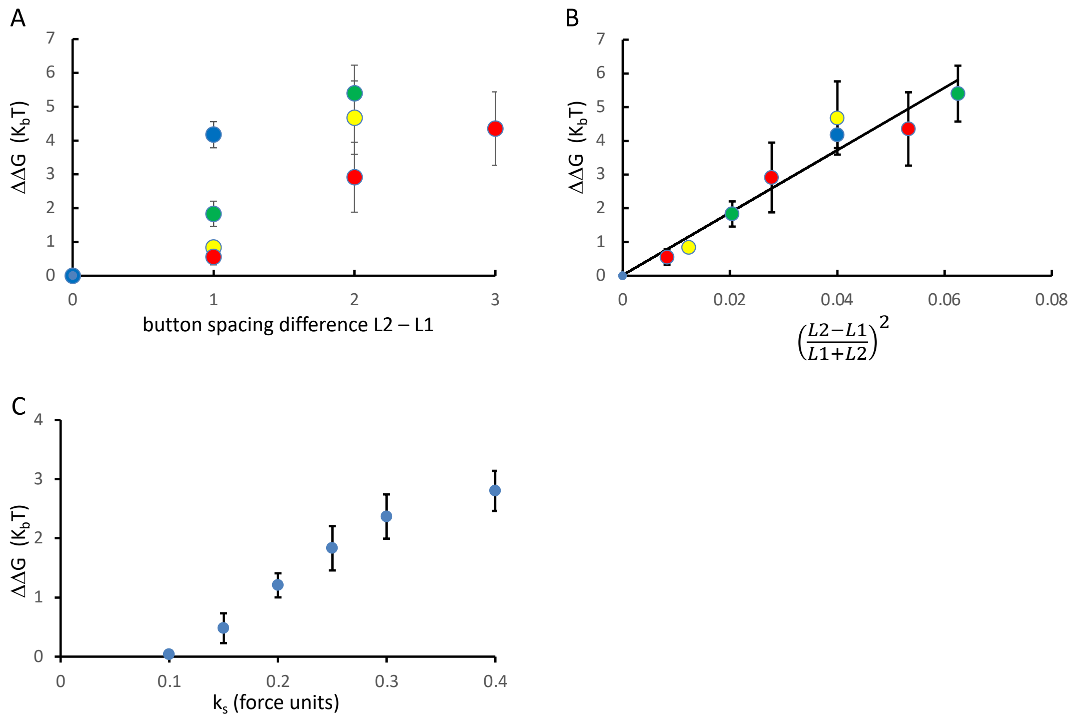


**Fig. S1. Energetics of barcode discrimination**. **A.** Difference in free energy between correct and incorrect associations as a function of the difference in spacing between pairs of buttons. Colors denote the spacing for one pair (L1) and the X axis denotes difference between the spacing of the other pair and the first pair (L2-L1). (blue) 2 links, (green) 3 links, (yellow) 4 links, (red) 5 links. Error bars denote standard deviation. **B.** Data from panel A re-plotted as a function of the squared normalized difference in button spacing. Line is a best-fit line to the data points. **C.** Difference in free energy between correct and incorrect associations as a function of the spring constant $k_s$ of the bead-spring chain model.

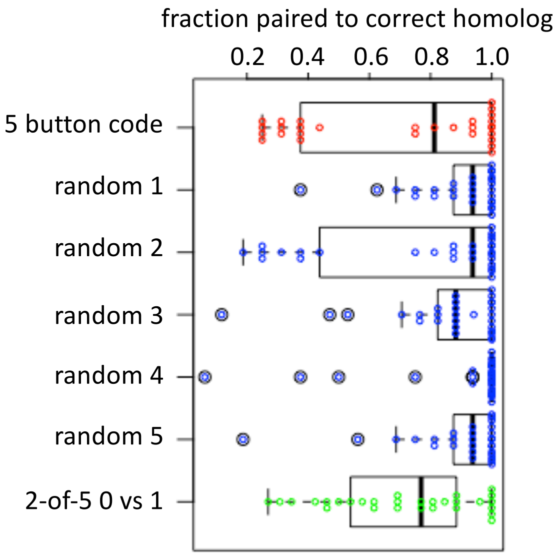


**Fig. S2.** Variations of button barcodes in which buttons and gaps are interchanged. Simulations were performed as in **Figures 5** and **6**, but using "inverted" barcodes in which the buttons were replaced by gaps and the gaps by buttons. The red datapoints correspond to the 5 button code shown in red in **Figure 5B**, but with each pairing button replaced with a non-pairing node, and each non-pairing node replaced with a pairing button. The blue datapoints correspond to the random 5 button codes in **Figure 5B** with each pairing button replaced with a non-pairing node, and each non-pairing node replaced with a pairing button. The green datapoints correspond to the 2-of-5 code example from **Figure 6** (0 vs 1) again with each pairing button replaced with a non-pairing node, and each non-pairing node replaced with a pairing button.

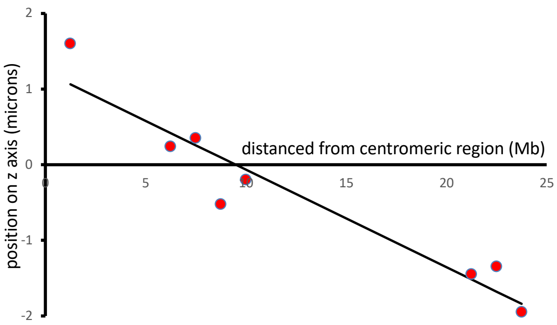


**Fig. S3.** Fitting z axis position to genomic position in *Drosophila* embryos supports an extended worm-like chain rather than a random coil at the length scale of the whole chromosome. Previously published three-dimensional FISH data from cycle 13 *Drosophila* embryos (Marshall 1996) were binned based on segment along chromosome arm 2L, and the average vertical distance (z) plotted as a function of the genomic distance of the segment from the centromeric region, in Mb. Best fit line is included with slope -1.3 microns per Mb. These data show that the chromosome arm spans a distance along the z axis of a little over 3 microns, and follows a linear relation of physical to genomic distance, consistent with an extended worm-like chain having a persistence length on the order of microns, but not consistent with a random coil in which case the spatial distance would scale as the square root of genomic distance.

## SI References


1. W. F. Marshall, J. C. Fung, Modeling meiotic chromosome pairing: nuclear envelope attachment, telomere-led active random motion, and anomalous diffusion. *Physical biology* **13**, 026003 (2016).

2. W. F. Marshall, J. C. Fung, Modeling meiotic chromosome pairing: a tug of war between telomere forces and a pairing-based Brownian ratchet leads to increased pairing fidelity. *Physical biology* **16**, 046005 (2019).

3. J. L. Sikorav, G. Jannink, Kinetics of chromosome condensation in the presence of topoisomerases: a phantom chain model. *Biophys J* **66**, 827–837 (1994).

4. B. Kundukad, J. R. C. van der Maarel, Control of the flow properties of DNA by topoisomerase II and its targeting inhibitor. *Biophys J* **99**, 1906–1915 (2010).

5. E. J. Navarro, W. F. Marshall, J. C. Fung, Modeling cell biological features of meiotic chromosome pairing to study interlock resolution. *PLoS Comput Biol* **18**, e1010252 (2022).

6. D. E. Comings, Arrangement of chromatin in the nucleus. *Hum Genet* **53**, 131–143 (1980).

7. W. F. Marshall, A. F. Dernburg, B. Harmon, D. A. Agard, J. W. Sedat, Specific interactions of chromatin with the nuclear envelope: positional determination within the nucleus in Drosophila melanogaster. *Mol Biol Cell* **7**, 825–842 (1996).

8. A. F. Dernburg, *et al.*, Perturbation of nuclear architecture by long-distance chromosome interactions. *Cell* **85**, 745–759 (1996).

9. J. Brickner, Genetic and epigenetic control of the spatial organization of the genome. *Mol Biol Cell* **28**, 364–369 (2017).

10. Q. Sun, A. Perez-Rathke, D. M. Czajkowsky, Z. Shao, J. Liang, High-resolution single-cell 3D-models of chromatin ensembles during Drosophila embryogenesis. *Nat Commun* **12**, 205 (2021).

11. A. I. Grosberg, A. R. Khokhlov, *Statistical physics of macromolecules* (AIP Press, 1994).

12. R. Peters, Nucleo-cytoplasmic flux and intracellular mobility in single hepatocytes measured by fluorescence microphotolysis. *EMBO J* **3**, 1831–1836 (1984).

13. F. Erdel, M. Baum, K. Rippe, The viscoelastic properties of chromatin and the nucleoplasm revealed by scale-dependent protein mobility. *J Phys Condens Matter* **27**, 064115 (2015).

14. J. Speil, U. Kubitscheck, Single ovalbumin molecules exploring nucleoplasm and nucleoli of living cell nuclei. *Biochim Biophys Acta* **1803**, 396–404 (2010).

15. K. N. Dahl, S. M. Kahn, K. L. Wilson, D. E. Discher, The nuclear envelope lamina network has elasticity and a compressibility limit suggestive of a molecular shock absorber. *J Cell Sci* **117**, 4779–4786 (2004).

16. A. D. Stephens, E. J. Banigan, S. A. Adam, R. D. Goldman, J. F. Marko, Chromatin and lamin A determine two different mechanical response regimes of the cell nucleus. *Mol Biol Cell* **28**, 1984–1996 (2017).

17. A. Kaufmann, F. Heinemann, M. Radmacher, R. Stick, Amphibian oocyte nuclei expressing lamin A with the progeria mutation E145K exhibit an increased elastic modulus. *Nucleus* **2**, 310–319 (2011).

18. N. Zuela-Sopilniak, *et al.*, Measuring nucleus mechanics within a living multicellular organism: Physical decoupling and attenuated recovery rate are physiological protective mechanisms of the cell nucleus under high mechanical load. *Mol Biol Cell* **31**, 1943–1950 (2020).

19. A. Vahabikashi, *et al.*, Nuclear lamin isoforms differentially contribute to LINC complex-dependent nucleocytoskeletal coupling and whole-cell mechanics. *Proc Natl Acad Sci U S A* **119**, e2121816119 (2022).

20. B. Lim, T. Heist, M. Levine, T. Fukaya, Visualization of Transvection in Living Drosophila Embryos. *Molecular Cell* **70**, 287–296 (2018).

21. M. B. 6th Child, *et al.*, Live imaging and biophysical modeling support a button-based mechanism of somatic homolog pairing in Drosophila. *Elife* **10** (2021).

22. M. Jagannathan, R. Cummings, Y. M. Yamashita, A conserved function for pericentromeric satellite DNA. *Elife* **7** (2018).

23. M. Jagannathan, R. Cummings, Y. M. Yamashita, The modular mechanism of chromocenter formation in Drosophila. *Elife* **8** (2019).

24. L. D. Landau, E. M. Lifshi͡ts, A. Markovich. Kosevich, L. P., *Theory of elasticity*, 3rd English ed., rev.enl. by E.M. Lifshitz, A.M. Kosevich, and L.P. Pitaevskii. (Pergamon Press, 1986).

25. C. Bouchiat, *et al.*, Estimating the persistence length of a worm-like chain molecule from force-extension measurements. *Biophys J* **76**, 409–413 (1999).

26. D. Allais, *Bar Code Symbology* (Intermec Corp. Report, 1984).

27. C. K. Harmon, *Reading between the lines: an introduction to bar code technology*, Fourth edition. (Helmers Pub., 1989).
